# Engineered human hepatocyte organoids enable CRISPR-based target discovery and drug screening for steatosis

**DOI:** 10.1038/s41587-023-01680-4

**Published:** 2023-02-23

**Authors:** Delilah Hendriks, Jos F. Brouwers, Karien Hamer, Maarten H. Geurts, Léa Luciana, Simone Massalini, Carmen López-Iglesias, Peter J. Peters, Maria J. Rodríguez-Colman, Susana Chuva de Sousa Lopes, Benedetta Artegiani, Hans Clevers

**Affiliations:** 1 Hubrecht Institute, Royal Netherlands Academy of Arts and Sciences, Utrecht, The Netherlands; 2 Oncode Institute, Utrecht, The Netherlands; 3 Research Group Analysis Techniques in the Life Sciences, School of Life Sciences and Technology, Avans University of Applied Sciences, Breda, The Netherlands; 4 Molecular Cancer Research, Center for Molecular Medicine, University Medical Center Utrecht, Utrecht, The Netherlands; 5 The Princess Maxima Center for Pediatric Oncology, Utrecht, The Netherlands; 6 The Maastricht Multimodal Molecular Imaging Institute, Maastricht University, Maastricht, The Netherlands; 7 Department of Anatomy and Embryology, Leiden University Medical Center, Leiden, The Netherlands; 8 University Medical Center Utrecht, Utrecht, The Netherlands; 9 Present Address: Pharma, Research and Early Development of F. Hoffmann-La Roche Ltd, Basel, Switzerland

**Keywords:** Genetic engineering, Phenotypic screening, Stem-cell biotechnology, Mechanisms of disease, Cell biology

## Abstract

The lack of registered drugs for nonalcoholic fatty liver disease (NAFLD) is partly due to the paucity of human-relevant models for target discovery and compound screening. Here we use human fetal hepatocyte organoids to model the first stage of NAFLD, steatosis, representing three different triggers: free fatty acid loading, interindividual genetic variability (PNPLA3 I148M) and monogenic lipid disorders (*APOB* and *MTTP* mutations). Screening of drug candidates revealed compounds effective at resolving steatosis. Mechanistic evaluation of effective drugs uncovered repression of de novo lipogenesis as the convergent molecular pathway. We present FatTracer, a CRISPR screening platform to identify steatosis modulators and putative targets using *APOB*^*−/−*^ and *MTTP*^*−/−*^ organoids. From a screen targeting 35 genes implicated in lipid metabolism and/or NAFLD risk, FADS2 (fatty acid desaturase 2) emerged as an important determinant of hepatic steatosis. Enhancement of FADS2 expression increases polyunsaturated fatty acid abundancy which, in turn, reduces de novo lipogenesis. These organoid models facilitate study of steatosis etiology and drug targets.

## Abstract (web summary)

Organoid models of early liver disease aid target discovery and drug screening.

## Main

Nonalcoholic fatty liver disease (NAFLD) is a major cause of chronic liver disease worldwide, with >25% of the global population affected^1^. NAFLD is a progressive disease. Starting with the accumulation of fat within the liver, hepatic steatosis, it can develop into nonalcoholic steatohepatitis (NASH), eventually resulting in liver failure or cancer. NAFLD risk is associated with different factors including nutritional overload, genetic predisposition and genetic lipid disorders, as well as environmental factors^2^. In particular, dietary habits such as high caloric intake and high carbohydrate/fat consumption are key risks^3^. Indeed, NAFLD is epidemiologically associated with obesity, type 2 diabetes and metabolic syndrome features^4^. Interindividual differences in susceptibility can be partly explained by genetic variation. Genome-wide association studies have revealed multiple NAFLD risk loci^5^, with a single-nucleotide polymorphism (SNP) in the *PNPLA3* gene as one of the strongest links^6^. Monogenic disorders of lipid metabolism can also predispose to NAFLD^7,8^, with sometimes already severe phenotypes in pediatric patients^9^. These include familial hypobetalipoproteinemia (incidence 1:500–1:1,000)^10^, predominantly caused by *APOB* mutations, and abetalipoproteinemia (incidence <1:100,000)^11^, caused by *MTTP* mutations.

Despite its high and rising prevalence^12^, effective drug therapies for any stage of NAFLD are lacking. Recent drug failures in clinical trials, such as obeticholic acid for NASH therapy^13^, underscore the current complexity in combatting NAFLD. Most emphasis has been placed on tackling the more advanced and clinical NASH stage^14,15^, while targeting disease at an early stage could become of future importance. Accordingly, efforts are being taken to identify biomarkers of early NAFLD^16^. Therapies effective for steatosis are therefore of considerable interest in regard to minimization of liver damage and disease progression.

NAFLD biology and putative drug therapies have predominantly been studied in rodents using genetic, chemically induced or diet-driven models^17^. However, inherent interspecies differences, amongst others in their metabolism, complicate translation of these findings to humans. Animal models also do not represent scalable systems for rapid and high-throughput target discovery. More recently, relevant in vitro human models of NAFLD relying on primary cells or pluripotent stem cell-derived cells have emerged^18–22^, but their applicability to systematic drug screenings remains unproven. In addition, a drawback of these models is their lack of expansion capacity and the difficulty in genetic engineering, which hampers genetic screens for target discovery. Human fetal hepatocyte organoids represent a culture system that recapitulates key features of mature in vivo hepatocytes^23^, including lipid metabolism profiles (Supplementary Fig. 1a,b). Their long-term expansion capacity has enabled us to genetically engineer these organoids using various CRISPR approaches^24–26^.

Here, we explored these features to model the first stage of NAFLD. We modeled a variety of different steatosis triggers and studied their interplay: (1) free fatty acid (FFA) loading to approximate a Western diet, (2) interindividual genetic risk through modeling the top risk variant, PNPLA3 I148M and (3) monogenic lipid disorders through creation of *APOB* or *MTTP* knockout (KO) organoids. We performed drug screening and identified compounds effective at resolving steatosis across these different models, while highlighting that PNPLA3 I148M impairs drug response. Through drug treatment-based clustering analysis of whole transcriptomes, we identified common classes of drug action and noted putative adverse drug effects. This approach revealed repression of de novo lipogenesis (DNL) as the common mechanism of action of the effective steatosis-reducing compounds. Finally, we leveraged the *APOB*- and *MTTP*-mutant organoids to establish a CRISPR-based screening platform to identify steatosis modulators/targets and to evaluate NAFLD risk genes. This platform, termed FatTracer, identified FADS2 as a critical steatosis modulator. While FADS2 loss aggravated steatosis phenotypes, its overexpression resulted in steatosis reduction.

## Results

### FFA-induced steatosis organoids

**Fig. 1:**
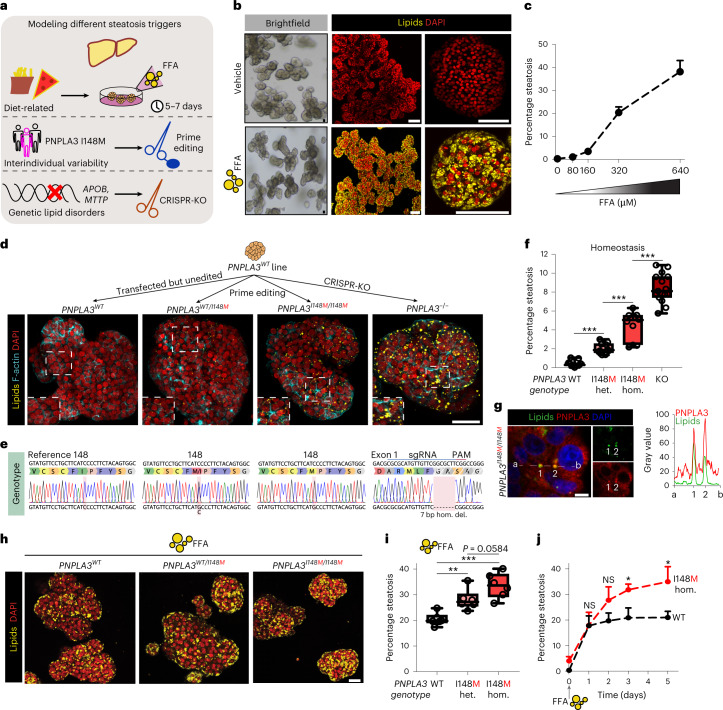
Precision gene editing in human fetal hepatocyte organoids evaluates genetic predisposition to steatosis. **a**, Strategy used to generate a panel of human hepatic steatosis organoid models. **b**, Brightfield images and Nile Red lipid staining of vehicle-treated and FFA-exposed (640 μM) WT organoids. **c**, Quantification of the percentage of steatosis after exposure to increasing FFA concentrations in WT organoids (mean ± s.d.). *n* = 6 independent replicates from two donors. **d**, Nile Red lipid staining overlaid with phalloidin of PNPLA3 variant organoids generated from the same donor. **e**, Sanger trace sequences of the *PNPLA3* genotypes of PNPLA3 variant organoids. **f**, Quantification of the percentage of spontaneous steatosis in PNPLA3 variant organoids. *n* = 11 independent replicates for *PNPLA3*^*WT*^ and *PNPLA3*^*WT/I148M*^, *n* = 10 independent replicates for *PNPLA3*^*I148M/I148M*^, *n* = 15 independent replicates for *PNPLA3*^*KO*^ from either two (WT, WT/I148M, I148M/I148M) or three (KO) clonal lines from two donors. Two-tailed nested *t-*test: KO versus WT and I148M/I148M versus WT, ***P < 0.0001; I148M/I148M versus WT/I148M, ***P = 0.0001. **g**, Left, immunofluorescence staining for PNPLA3 and lipid droplet visualization with BioTracker 488 Green Lipid Droplet Dye in *PNPLA3*^*I148M/I148M*^ organoids. Right, quantification of the fluorescence signal from a to b through lipid droplets (1 and 2). **h**, Nile Red lipid staining of FFA-exposed (320 μM) PNPLA3 variant organoids. **i**, Quantification of the percentage of steatosis following FFA exposure in PNPLA3 variant organoids. *n* = 6 independent replicates from two clonal lines from two donors per genotype. Two-tailed nested *t*-test: WT/148M versus WT, **P = 0.0026; I148M/I148M versus WT, ***P = 0.0002; I148M/I148M versus WT/148M, *P* = 0.0584. **j**, Quantification of the percentage of steatosis over time after FFA exposure (320 μM) in *PNPLA3*^*WT*^ and *PNPLA3*^*I148M/I148M*^ organoids generated from the same donor (mean ± s.d.). *n* = 3 independent replicates per genotype. Two-tailed *t*-test: day 3, *P = 0.0128; day 5, *P = 0.0191; NS, not significant. **f**,**i**, The box indicates the 25–75th percentiles, the center line indicates the median and the whiskers indicate minimum and maximum values. **b**,**d**,**g**,**h**, Representative of *n* = 10, 6, 3 and 6 independent experiments, respectively. Scale bars, 100 μm (**b**), 50 μm (**d**), 5 μm (**g**) and 25 μm (**h**). PAM, protospacer adjacent motif; hom., homozygous; het., heterozygous; del., deletion.

Different and multiple causes underlie the development of hepatic steatosis. We set out to develop models that could capture both exogenous and genetic triggers of human hepatic steatosis using human fetal hepatocyte organoids (Fig. 1a). Of note, characterization of the genotypes of the four different organoid lines used throughout this study revealed all donors to not be carriers of three well-established coding NAFLD risk SNPs (PNPLA3 I148M, TM6SF2 E167K and GCKR P446L), with the exception of one donor being a heterozygous carrier of GCKR P446L (Supplementary Fig. 1c and Supplementary Table 1). The hallmark feature of steatosis is triglyceride (TAG) accumulation. Potential sources contributing to the hepatic fatty acid pool for TAG synthesis include (1) dietary fatty acids taken up in the form of chylomicron remnants from the small intestine; (2) the plasma FFA pool comprising those derived from lipolysis of adipose tissue as well as those from spillover of lipoprotein lipase-generated FFAs from chylomicrons; and (3) hepatic DNL using carbohydrate sources^27^. Serum FFAs are the main contributor to the hepatic fatty acid pool and are elevated in NAFLD patients, strongly linked to dietary and lifestyle habits^27,28^. We thus defined a FFA-induced steatosis model by challenging wild-type (WT) organoids with a concentrated mixture of oleic acid and palmitic acid (1:1 ratio), two of the most abundant FFAs in the plasma pool^29^. While at low concentrations organoids were able to process externally provided FFAs, at higher concentrations hepatocytes progressively accumulated lipids, as visualized by Nile Red staining (Fig. 1b and Supplementary Fig. 2a). Quantification of the steatosis level (defined as the area of lipid droplet coverage within an organoid) revealed a progressive induction of steatosis, reaching >40% at the highest FFA challenge (Fig. 1c). Concomitant with the progressive development of steatosis, the organoids displayed impaired proliferation capacity as confirmed by a sharp reduction in Ki-67^+^ cells (Supplementary Fig. 2a,b), which may be explained by the lipotoxic effects of certain fatty acids, especially saturated ones such as palmitic acid present in the FFA mixture^30^. FFA exposure reproducibly induced similar levels of steatosis in organoids from different donors (Supplementary Fig. 2c).

### Personalized PNPLA3 organoids

We next focused on modeling genetic predisposition to NAFLD. The rs738409 variant in the *PNPLA3* gene encoding the I148M variant is the strongest genetic risk factor to date^6^. Mouse studies have provided important indications into its role, such as alterations in TAG metabolism following overexpression of the human I148M variant^31^. A recent study repopulating mouse livers with human hepatocytes, either from WT (I148I/I148I) or homozygous I148M donors, found that the I148M variant exacerbated steatohepatitis^32^. We turned to diverse CRISPR approaches to generate a complete panel of isogenic human PNPLA3 variant organoids. We used prime editing^33^ to introduce the I148M variant in organoids from donors with a I148I/I148I genotype to generate both heterozygous (*PNPLA3*^*WT/I148M*^) and homozygous (*PNPLA3*^*I148M/I148M*^) variant organoids, while similarly transfected but unedited organoids (*PNPLA3*^*WT*^) were used as internal controls, thereby covering all possible genotypes (Supplementary Fig. 3a). Within this isogenic setting, hepatocytes from two different donors were efficiently prime edited (Fig. 1d,e and Supplementary Fig. 3b). We also generated *PNPLA3* KO (*PNPLA3*^*−/−*^) organoids using conventional CRISPR–Cas9 (ref. ^24^) for comparative analysis (Fig. 1d,e and Supplementary Fig. 3a), and confirmed the loss of PNPLA3 protein as assessed by antibody staining (Supplementary Fig. 3c).

The *PNPLA3* genotype directly influenced lipid levels within hepatocytes. As expected, *PNPLA3*^*WT*^ organoids do not accumulate lipids in homeostasis (that is, under standard culture conditions). Instead, all three engineered *PNPLA3* genotypes spontaneously yielded lipid phenotypes (Fig. 1d and Supplementary Fig. 3d). *PNPLA3*^*−/−*^ organoids most extensively accumulated lipids (around 8% steatosis), followed by homozygous I148M organoids (around 5%) and heterozygous I148M organoids (around 2%) (Fig. 1f). Lipid phenotypes were consistent across all generated clonal lines for the different *PNPLA3* genotypes (Fig. 1f and Supplementary Fig. 3e). The accumulation of PNPLA3 protein on lipid droplets has been proposed as a mechanism underlying the increased steatosis risk in I148M carriers^34^. In *PNPLA3*^*I148M/I148M*^ organoids, we found that many of the small lipid droplets colocalized with increased intensity of PNPLA3 protein signal (Fig. 1g and Supplementary Fig. 3f).

We next interrogated the interplay between *PNPLA3* genotypes and FFA-induced steatosis. Both *PNPLA3*^*WT/I148M*^ and *PNPLA3*^*I148M/I148M*^ organoids displayed increased sensitivity to a mild FFA challenge (320 μM), developing more severe steatosis, as compared with their *PNPLA3*^*WT*^ counterparts (Fig. 1h). Similar evidence on homozygous PNPLA3 I148M was recently observed in two-dimensional pluripotent stem cell-derived hepatocytes^22^. Quantification of steatosis levels following FFA challenge confirmed these observations and indicated a trend of more severe steatosis in the I148M variant in homozygosity as compared with that in heterozygosity (Fig. 1i). We then evaluated the time-dependent effects of steatosis induction in homozygous I148M organoids in relation to their WT counterparts, to understand the dynamics of the aggravated steatosis phenotype. While after 1 day of FFA exposure both PNPLA3 variant organoids presented with a similar steatosis degree, fat levels were consistently elevated in *PNPLA3*^*I148M/I148M*^ organoids from day 2 onwards (Fig. 1j). This may indicate that the I148M variant impairs the capacity of hepatocytes to secrete/hydrolyze lipids or, alternatively, that the balance between de novo synthesis and lipid disposal is perturbed. Changes in the capacity of lipid particle assembly and export have been reported in I148M carriers^35,36^, and mouse studies likewise have hinted at an impairment in TAG hydrolysis^31^.

Altogether, these comparative studies on the different *PNPLA3* genotypes engineered at the endogenous locus suggest that the I148M variant phenocopied—but to a lesser extent—the effect of PNPLA3 loss. KO of the *Pnpla3* gene in mice does not induce steatosis^37^ and therefore depletion of PNPLA3 has been suggested as a putative clinical approach^34,38^. Our findings challenge this idea, because complete loss of PNPLA3 protein in human hepatocytes induces steatosis (Fig. 1d–f), highlighting important interspecies differences.

### Genetic steatosis organoids

**Fig. 2:**
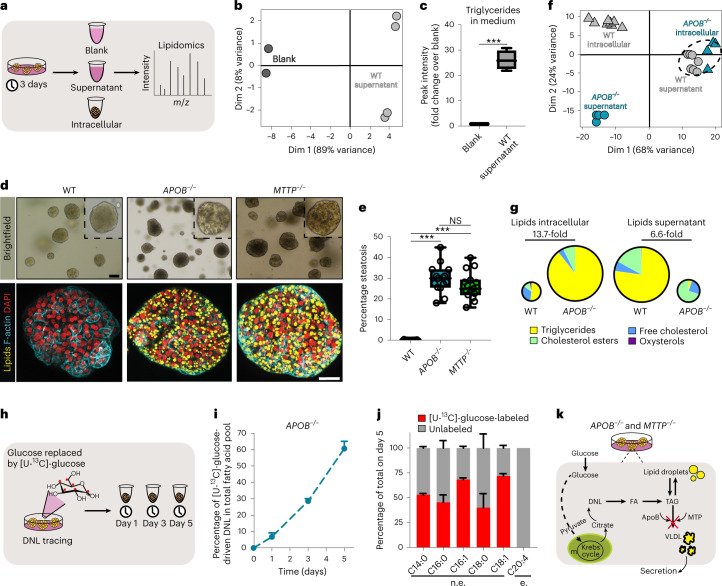
Organoid models of DNL-generated lipid accumulation by introduction of *APOB* or *MTTP* mutations. **a**, Workflow used to perform lipidomics on organoid cultures. **b**, PCA on neutral lipids found in the supernatant of WT organoids and in blank medium. *n* = 4 independent measurements in WT organoid cultures from two donors; *n* = 2 independent measurements for blank medium. **c**, Quantification of TAG content in the supernatant of WT organoids relative to blank medium. Sample sizes as in **b**. Two-tailed *t*-test: ***P = 0.0008. **d**, Brightfield images and Nile Red lipid staining overlaid with phalloidin of WT, *APOB*^*−/−*^ and *MTTP*^*−/−*^ organoids. Representative of *n* = 9 independent experiments. **e**, Quantification of the percentage of spontaneous steatosis in WT, *APOB*^*−/−*^ and *MTTP*^*−/−*^ organoids. *n* = 15 independent replicates from three clonal lines per genotype from three donors. Two-tailed nested *t*-test: *APOB*^*−/−*^ versus WT, ***P < 0.0001; *MTTP*^*−/−*^ versus WT, ***P = 0.0002. **f**, PCA of neutral lipids found intracellularly and in the supernatant of *APOB*^*−/−*^ and WT organoids. *n* = 4 independent measurements in *APOB*^*−/−*^ organoid cultures from two donors; *n* = 8 independent measurements in WT organoid cultures from the same two donors. **g**, Pie charts indicating the average distribution of different neutral lipid species in WT and *APOB*^*−/−*^ organoids. Pie size reflects average FC in total lipid amount. Sample sizes as in **f** (see also Supplementary Fig. 6c). **h**, Workflow used to perform DNL tracing using [U-^13^C]-glucose in *APOB*^*−/−*^ organoids. **i**, Quantification of the percentage of glucose-driven DNL contribution to the fatty acid pool in *APOB*^*−/−*^ organoids (mean ± s.d.). *n* = 2 independent quantifications in *APOB*^*−/−*^ organoid cultures from two donors. **j**, Quantification of the percentage of glucose-driven DNL contribution for five nonessential (n.e.) fatty acids in *APOB*^*−/−*^ organoids 5 days post tracing (mean ± s.d.). No labeling is observed for the essential (e.) fatty acid C20:4. Sample size as in **i**. **k**, Mechanism of lipid accumulation in *APOB*^*−/−*^ and *MTTP*^*−/−*^ organoids. **c**,**e**, The box indicates the 25–75th percentiles, the center line indicates the median and the whiskers indicate minimum and maximum values. **d**, Scale bars, 100 μm (brightfield) and 25 μm (fluorescence). Dim, dimension.

We next aimed to model two monogenic lipid disorders that predispose to NAFLD: familial hypobetalipoproteinemia and abetalipoproteinemia^7,8^. These diseases most commonly result from monoallelic or, more rarely, biallelic, loss of *APOB* and *MTTP*, respectively. Their gene products, ApoB and MTP, are critically involved in the packaging of TAG into very-low-density lipoprotein (VLDL) particles. The resulting impaired VLDL secretion in turn confers susceptibility to steatosis. We first addressed the VLDL secretory capacity of WT organoids. To this end, we cultured organoids for 3 days without changing the culture medium, collected the supernatant and performed lipidomics on neutral lipids (Fig. 2a). Principle component analysis (PCA) revealed distinct clustering of the lipid profiles of blank medium (which contained very few lipid species (Supplementary Fig. 4a)) versus the medium in which WT organoids were maintained (Fig. 2b). Indeed, TAG content in the medium was enriched by approximately 25-fold (Fig. 2c and Supplementary Fig. 4a,b), confirming that WT organoids actively secrete VLDL particles.

We then proceeded to CRISPR-engineer WT organoids to generate *APOB-* and *MTTP-*mutants (Supplementary Fig. 5a). When hepatocytes were targeted with either APOB-single-guide RNA (sgRNA)/Cas9 or MTTP-sgRNA/Cas9, we immediately noticed distinct outgrowth of darker organoids (Supplementary Fig. 5b). We derived clonal lines from these organoids, which presented with many lipid droplets visible under brightfield microscopy (Fig. 2d). Lipid accumulation in these lines occurred spontaneously under homeostatic conditions, without any exogenous stimulus. Genotyping and immunofluorescence staining revealed the presence of homozygous *APOB* or *MTTP* mutations and concomitant loss of protein expression, respectively (Supplementary Fig. 5c–f). Of note, heterozygous *APOB*^*+/−*^ organoids accumulated only few lipid droplets (Supplementary Fig. 5g). Nile Red staining confirmed extensive lipid accumulation within *APOB*^*−/−*^ and *MTTP*^*−/−*^ organoids (Fig. 2d), reaching a steatosis level of around 30% for both mutants (Fig. 2e). Transmission electron microscopy further visualized the abundant presence of lipid droplets of various size throughout the cytoplasm, as well as in the nucleus, of *APOB*^*−/−*^ organoids (Supplementary Fig. 5h). In addition, while in WT organoids VLDL particles were readily detectable in typical vesicles around the Golgi apparatus (Supplementary Fig. 5h), these were completely absent in *APOB*^*−/−*^ organoids, further validating the consequences of *APOB* KO in the organoids. We confirmed these lipid phenotypes by generating *APOB*- and *MTTP*-mutants from multiple donors (Supplementary Fig. 6a,b). Despite their extensive steatosis, hepatocyte proliferation capacity was unaffected (Supplementary Fig. 6c), contrasting with the FFA-induced steatosis model. This suggested that different underlying sources of lipid accumulation induce distinct cell responses. In line, it has been recognized that not all lipid species are equally toxic^39^.

We reasoned that DNL should be the source of lipid accumulation in *APOB*^*−/−*^ and *MTTP*^*−/−*^ organoids, because the culture medium itself contains minimal lipid sources and only small amounts of essential fatty acids (that is, those that cells cannot synthesize). Culturing these mutants in the complete absence of the potential confounding lipid sources (for example, RSPO1-conditioned medium) accordingly did not alter the steatosis phenotypes (Supplementary Fig. 4c). To directly probe the origin of the accumulated lipids and to assess changes in lipid profiles, we subjected both *APOB*^*−/−*^ and WT organoids to lipidomic analyses, this time interrogating both the intracellular lipid profiles (organoid pellets) and secreted lipid profiles (supernatants) (Fig. 2a). Notably, PCA revealed distinct clustering where the intracellular lipid profiles of *APOB*^*−/−*^ organoids closely clustered with the supernatants of WT organoids, confirming defective lipid export in ApoB mutants (Fig. 2f and Supplementary Fig. 4a). Quantification of the lipid content corroborated the extensive lipid accumulation within *APOB*^*−/−*^ organoids (about 14-fold compared with WT), with the predominant change being extensive TAG accumulation, while conversely secreted lipids (especially TAG) in the supernatant were nearly absent (Fig. 2g and Supplementary Fig. 4d–g). To address the contribution of DNL to the observed lipid profiles, we performed a time-resolved [U-^13^C]-glucose isotope-tracing experiment (Fig. 2h). We calculated the incorporation of labeled glucose into the synthesis of five of the most abundant fatty acids (C14:0, C16:0, C16:1, C18:0 and C18:1) which, together, constitute >90% of the total intracellular fatty acid pool and >85% of the fatty acid pool that can be synthesized by DNL. We observed a time-dependent increase in [U-^13^C]-glucose-dependent DNL contribution, which linearly increased to approximately 60% on day 5, with a low extent of heterogeneity between the contribution of the individual fatty acids (Fig. 2i–j). The validity of this approach was confirmed by the absence of labeling of essential fatty acids that cannot be synthesized by human cells (for example, C20:4) (Fig. 2j and Supplementary Fig. 4h). A 100% incorporation would not be expected, because other sources contributing to the acetyl-CoA pool, such as amino acids and culture medium-provided pyruvate and glutamine, were not traced in this setting. Thus, glucose-driven DNL is the main contributor to the spontaneous steatosis phenotype of these lipid secretion-defective organoids (Fig. 2k). Both *APOB*^*−/−*^ and *MTTP*^*−/−*^ mutants constitute natural steatosis organoid models that can, like their WT counterparts^23,24^, be long-term expanded in culture for at least 2 years with stable steatosis levels (Supplementary Fig. 6d).

### Selected drug candidates reduce steatosis

**Fig. 3:**
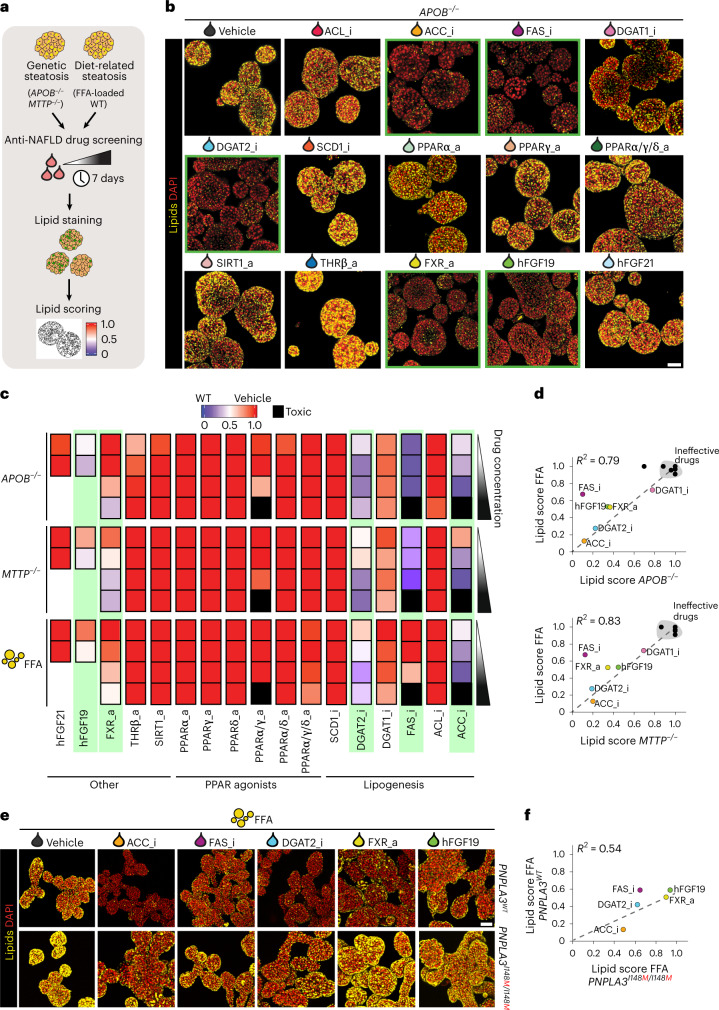
Capturing drug responses in different organoid models of human hepatic steatosis. **a**, Workflow used to perform NAFLD drug screening in the different steatosis organoid models. **b**, Nile Red lipid staining of *APOB*^*−/−*^ organoids after treatment with the different indicated drugs. Green-bordered boxes highlight steatosis-reducing effects. Representative of *n* = 4 independent experiments using two donors. **c**, Lipid score analyses of genetic steatosis (*APOB*^*−/−*^ and *MTTP*^*−/−*^ organoids) and FFA-induced steatosis (FFA-exposed WT organoids) following treatment with the different indicated drugs. Each square represents a concentration, with four increasing concentrations as per the white-to-black triangle; see Supplementary Table 2 for drug concentrations. Black squares indicate drug toxicity (Supplementary Fig. 7e); green boxes highlight effective drugs. An arbitrary color scale ranging from 0 (blue, representing the lipid level in WT organoids) to 1 (red, representing vehicle-treated steatosis organoids from each model) is used. Quantifications represent the average lipid score from *n* = 3 organoid cultures/drug concentration. **d**, Correlation plots between lipid scores of *APOB*^*−/−*^ organoids (top) and *MTTP*^*−/−*^ organoids (bottom) versus those of FFA-exposed WT organoids following treatment with the different indicated drugs at the highest drug concentration, except for ACC_i, FAS_i and PPARα/γ_a, for which the third-highest dose is depicted. The coefficient of determination (*R*^2^) is indicated. **e**, Nile Red lipid staining of *PNPLA3*^*WT*^ and *PNPLA3*^*I148M/I148M*^ organoids (generated from the same donor) after treatment with selected drugs under FFA-induced steatosis. The most effective drug concentration (as used in **d**) was used. Representative of *n* = 2 independent experiments. **f**, Correlation plots between lipid scores of *PNPLA3*^*I148M/I148M*^ organoids versus those of *PNPLA3*^*WT*^ organoids following treatment with the selected drugs. Quantifications represent average lipid score from *n* = 3 organoid cultures/drug. *R*^2^ is indicated. **b**,**e**, Scale bars, 100 μm.

Multiple putative NAFLD drugs are currently under evaluation in clinical trials^40,41^, largely based on encouraging results from rodent studies. Addressing drug effects on different NAFLD stages and identifying the mechanism of action on a particular cell type is difficult to interpret from in vivo studies. We reasoned that our developed models could constitute screening platforms to identify drugs that directly act in hepatocytes to reduce steatosis, and could also address whether drug responses differ depending on the steatosis trigger (that is, FFA-driven versus DNL-driven). We selected 17 candidate NAFLD drugs from recent drug development programs and from new targets reported in the literature, focusing on those with putative hepatic effects^40,41^ (Supplementary Fig. 7a and Supplementary Table 2), and screened these drugs in the FFA-induced and genetic (*APOB*^*−/−*^ and *MTTP*^*−/−*^) steatosis models. For the FFA model, WT organoids were first made steatotic with a fixed concentration of FFAs (500 μM) for 3 days before drug administration, to induce a degree of steatosis similar to that observed in *APOB*^*−/−*^ and *MTTP*^*−/−*^ organoids (Supplementary Fig. 8a–c). We then exposed these models to the drugs (for the FFA-induced steatosis model, still in the presence of FFAs) for 7 days. Lipid accumulation was assessed through Nile Red staining, and we developed a lipid droplet scoring system to score drug effectiveness (Fig. 3a and Supplementary Fig. 7b).

A selected set of drugs markedly reduced the steatosis phenotypes of both *APOB*^*−/−*^ and *MTTP*^*−/−*^ organoids (Fig. 3b), most with a dose-dependent effect (Supplementary Fig. 7c). Effective drugs included inhibitors of ACC, FAS and DGAT2, a FXR agonist, as well as recombinant hFGF19. Using live imaging of *APOB*^*−/−*^ organoids following treatment with ACCi we could trace drug effects in real time, which revealed an extensive reduction in lipid content within the first 72 h (Supplementary Fig. 7d and Supplementary Video 1). We then evaluated drug responses in the FFA-induced steatosis model, noting a very similar drug response pattern as compared with the genetic models (Supplementary Fig. 8d).

We quantitatively assessed drug responses across the different models using our lipid scoring system to rank drug effectiveness (Fig. 3c). This confirmed a strong correlation between the FFA and genetic models (*R*^2^ = 0.79 and 0.83 for *APOB*^*−/−*^ and *MTTP*^*−/−*^, respectively) (Fig. 3d). Many of the identified effective targets act in hepatic lipogenesis (Fig. 3c,d). The strongest effects were observed following inhibition of acetyl-CoA carboxylase (ACC), converting acetyl-CoA to malonyl-CoA, which reduced steatosis levels to near WT organoid level. Inhibition of diacylglycerol O-acyltransferase 2 (DGAT2), which catalyzes the final reaction of TAG synthesis, also strongly reduced steatosis in all models. Finally, inhibition of fatty acid synthase (FAS), which converts acetyl-CoA and malonyl-CoA to palmitate, was also effective in both systems but with stronger effects in the genetic steatosis models. Nevertheless, other lipogenic targets were ineffective, suggesting an unprecedented high target dependency within the lipogenesis pathway (Fig. 3c,d). In fact, inhibition of ATP citrate lyase (ACL), which acts at the intersection of carbohydrate and lipid metabolism by conversion of citrate to acetyl-CoA, was ineffective, as was inhibition of stearoyl-CoA desaturase 1 (SCD1), which catalyzes the synthesis of monounsaturated fatty acids. Additionally, we noted only a very modest steatosis-reducing effect of DGAT1 inhibition in both genetic and FFA models, contrasting the effects of DGAT2i. DGAT1 primarily esterifies exogenous fatty acids while DGAT2 preferentially acts on DNL-derived fatty acids^42^, potentially underlying these differences. Combined DGAT1 and DGAT2 inhibition provided a synergistic effect that completely reverted the steatosis phenotypes of both genetic and FFA-induced steatosis models, already evident at nanomolar concentrations (Supplementary Fig. 9a–e).

Beyond beneficial effects on certain lipogenic enzymes, treatment with the FXR activator (FXRa) also induced a marked steatosis reduction with a similar extent in both genetic and FFA models (roughly 50%) (Fig. 3c,d). In mice, FXR activation imposes lipid-reducing effects in the liver, amongst others through reduced lipid absorption in the intestine^43^. Nevertheless, direct effects of FXR activation in the liver have also not been ruled out^43^. Our data demonstrate that FXRa poses direct steatosis-reducing effects in hepatocytes. Lastly, we observed a discrepancy between hFGF19 and hFGF21 treatment. Both have shown promising results for treatment of NASH, but whether their effects are mediated through direct or indirect actions on the liver is unresolved^44^. We found that recombinant hFGF19 effectively reduced steatosis, under both genetic and FFA-induced steatosis (about 50%), while hFGF21 failed to do so (Fig. 3c,d). The remainder of the screened drugs did not display steatosis-reducing effects in either of the models. Notably, none of the selective/dual/pan-peroxisome proliferator-activated receptor (PPAR) agonists influenced steatosis levels in a hepatocyte-directed fashion. Of note, PPAR agonists limit macrophage and stellate cell activation in the context of NASH in mice^45^, suggesting beneficial effects at more advanced disease stages. We further found no evidence of steatosis reduction following thyroid receptor-beta agonism and sirtuin 1 activation (Fig. 3c).

### PNPLA3 I148M influences drug response

We then interrogated whether genetic predisposition would influence drug treatment responses. We evaluated the five steatosis-reducing drugs (ACCi, FASi, DGAT2i, FXRa and hFGF19) in our *PNPLA3*^*WT*^ and *PNPLA3*^*I148M/I148M*^ organoids in a FFA-induced steatosis setting. As observed earlier (Fig. 1h–i), vehicle-treated I148M homozygous organoids displayed a worsened steatosis phenotype in response to the same FFA concentration as compared with their isogenic WT counterparts (Fig. 3e). Inhibition of ACC, FAS or DGAT2 posed obvious steatosis-reducing effects (Fig. 3e). Unexpectedly, we observed a diminished response towards both FXRa and hFGF19 treatment (approximately 15% steatosis reduction) which, in *PNPLA3*^*WT*^ organoids, reduced steatosis instead by roughly 50% (Fig. 3c–e). We calculated lipid scores and correlated them with those observed in *PNPLA3*^*WT*^ organoids (*R*^2^ = 0.54). This highlighted the generally decreased effect of drug treatment in organoids carrying the I148M variant (Fig. 3f), with the most obvious differences following treatment with FXRa and hFGF19. Our proof-of-concept data implied that, at similar levels of fat challenge, drug effectivity could be compromised by genetic risk factors.

### Drug screening using PLIN2 lipid reporter organoids

Perilipins coat lipid droplet particles, with PLIN2 being most abundantly expressed in the liver^46^. We hypothesized that fluorescence labeling of lipid droplets by tagging the endogenous *PLIN2* locus might enable the development of an imaging-based steatosis drug-screening system. To this end, we used our nonhomologous end-joining-mediated gene knock-in strategy^25^ (Supplementary Fig. 10a). *PLIN2* could be efficiently tagged with tdTomato or mNEON in both *APOB*^*−/−*^ and *MTTP*^*−/−*^ organoids (Supplementary Fig. 10b). The fluorescent signal derived from PLIN2 reporters was of typical lipid droplet morphology (Supplementary Fig. 10c). Counterstaining of the reporters with a lipid droplet dye confirmed faithful tagging of lipid droplets (Supplementary Fig. 10d), and tagging of PLIN2 did not alter steatosis phenotypes (Supplementary Fig. 10e). As a proof of principle, we treated the tagged organoids with ACCi which, as expected, reduced the endogenous PLIN2 fluorescent signal (Supplementary Fig. 10f). We next subjected the organoids to a set of five previously screened drugs (three positive, two negative) to assess the robustness of the system. Quantification of the fluorescent signal over a timeframe of 7 days visualized variable drug dynamics with time and demonstrated an identical classification of effective drugs (Supplementary Fig. 10g,h). Thus, PLIN2-reporter organoids act as real-time lipid reporter systems, providing the possibility of higher throughput and semiautomated analysis of drug effects on steatosis.

### Distinct drug effects on global hepatocyte transcriptomes

**Fig. 4:**
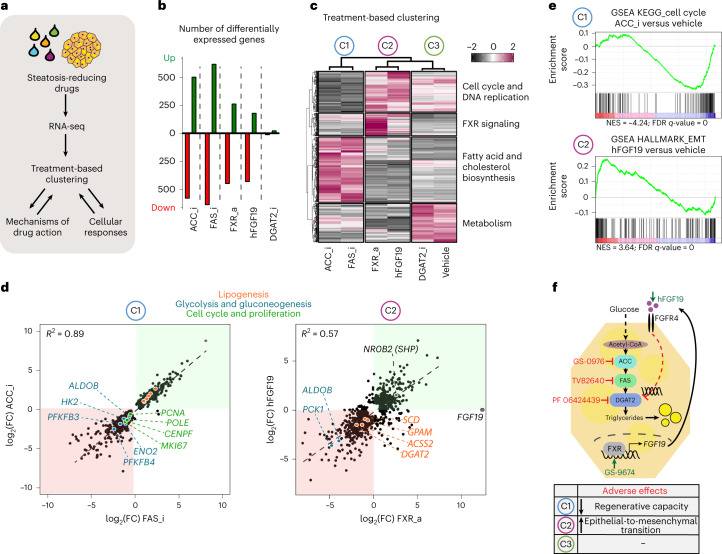
Transcriptome-based treatment clustering reveals steatosis-reducing drug actions and adverse effects. **a**, Workflow used to address the drug effects of steatosis-reducing drugs at the whole-transcriptomic level. **b**, Number of DEGs following different drug treatments relative to vehicle-treated *APOB*^*−/−*^ organoids (|log_2_(FC > 0.5)|, *P* < 0.005 (Wald test)). Whole-transcriptome analysis is based on sequencing of *n* = 2 *APOB*^*−/−*^ lines from two donors treated with the indicated drugs or vehicle. **c**, Treatment-based clustering and assignment of biological processes per gene cluster based on all DEGs identified across the different drug treatments. The heatmap depicts representative gene expression levels of one donor in response to the indicated drugs. Similar results were obtained for the other donor. Row *z*-scores are plotted. **d**, Correlation plots between the DEGs (|log_2_(FC > 0.5)|, *P* < 0.005 (Wald test) in at least one drug treatment) identified in C1 (ACC_i and FAS_i) (left) and C2 (hFGF19 and FXR_a (right)). Green and red boxes highlight common upregulated and downregulated genes, respectively. Selected DEGs belonging to key pathways are highlighted in specific colors as indicated. *R*^2^ is indicated. **e**, Gene set enrichment analysis for genes related to the cell cycle for C1 using ACC_i (*n* = 125 genes), and for genes related to epithelial-to-mesenchymal transition for C2 using hFGF19 (*n* = 200 genes) in comparison with vehicle-treated *APOB*^*−/−*^ organoids. Normalized enrichment scores (NES) and associated false discovery rate (FDR) *q*-values are indicated. **f**, Schematic of the proposed mechanism of action of effective steatosis-reducing drugs. The table indicates identified adverse drug effects per treatment cluster.

We next investigated drug responses and effects at the whole-transcriptome level to evaluate drug mechanisms, but also to assess overall cellular responses including, for example, adverse effects. We subjected *APOB*^*−/−*^ organoids treated with the effective drugs (DGAT2i, FASi, ACCi, FXRa, or hFGF19) or vehicle to bulk RNA-sequencing (RNA-seq) (Fig. 4a). Despite the fact that the net effect of all the different treatments was the same (that is, reduction of steatosis), differential gene expression analysis showed very different extents of transcriptomic rewiring. ACCi and FASi resulted in the highest number of differentially expressed genes (DEGs) (around 1,000 genes), followed by FXRa and hFGF19 (around 700 genes), while DGAT2i hardly affected the transcriptome (Fig. 4b). We then performed unsupervised clustering analysis based on the DEGs. This revealed three distinct treatment-based clusters: C1 comprising ACCi and FASi, C2 comprising FXRa and hFGF19 while C3 was composed of DGAT2i that clustered with vehicle treatment (Fig. 4c). The DEGs could be grouped into four main classes related to specific biological processes, which highlighted that the main changes within C1, C2 and C3 concerned metabolic pathways and the cell cycle.

### Treatment-based clusters reveal drug action and side effects

We zoomed into the transcriptomic changes per treatment cluster. C1 and C3 consisted of inhibitors directly targeting key enzymes of DNL. However, only in C1 did we observe marked transcriptomic changes. Within C1, ACCi- and FASi-treated organoids highly correlated (*R*^2^ = 0.89) (Fig. 4d). We observed downregulation of several key genes encoding proteins involved in glycolysis and gluconeogenesis (for example, *ALDOB*, *HK2*, *ENO2*, *PFKFB3/4*). Because inhibition of ACC or FAS proteins probably leads to a buildup of acetyl-CoA, this response likely constitutes a cellular coping mechanism by which pathways driving acetyl-CoA production are in turn repressed. We additionally observed a counterintuitive induction of many genes encoding lipogenic proteins (*SREBF1*, *ACACA*, *FASN* and so on) in C1 (Supplementary Fig. 11a). A similar observation was reported in hepatocyte-specific *Acaca/Acacb* KO mice^47^. This presumably represented an overcompensation effect due to the consequential lack of certain fatty acid species following blocked ACC or FAS activity. Possibly as a consequence of this, we also noted a strong repression of many genes encoding proteins involved in the cell cycle and DNA synthesis (for example, *MKI67*, *PCNA*, *BUB2*, *POLE*) (Fig. 4d,e and Supplementary Fig. 11b), indicating decreased hepatocyte proliferation and, in a broader context, impaired liver regeneration. These overall changes thus highlighted potential important adverse effects of these two drugs.

FXRa and hFGF19 constituted cluster C2, whose transcriptomic responses displayed substantial correlation (*R*^2^ = 0.57) (Fig. 4d). *FGF19* is primarily known as an intestinal FXR target gene^48^. Given the strong global transcriptomic similarities, we evaluated possible crosstalks between the two treatments. Crucially, FXRa treatment—but not hFGF19 treatment—strongly induced hepatocyte *FGF19* expression (Fig. 4d). We thus hypothesized that direct beneficial effects on steatosis of FXR activators in hepatocytes are primarily mediated through induction of hepatic FGF19 signaling. In agreement with FXR function, typical bile acid synthesis-related FXR transcriptional responses were detected following FXRa treatment (including repression of *CYP7A1* and induction of *NR0B2* (encoding small heterodimer partner, SHP)), but notably also following hFGF19 treatment (Supplementary Fig. 12a), suggesting indeed that many of the downstream effects of FXRa are the consequence of active FGF19 signaling. Additionally, we noted only a few DEGs unique to either condition (Supplementary Fig. 12b). We next searched for hints in the transcriptomic changes to explain how activation of the FXR–FGF19 signaling axis in hepatocytes could ultimately reduce steatosis. The repression of key genes encoding proteins involved in (de novo) lipogenesis—including for example *DGAT2* and *GPAM*—caught our attention. In addition, we observed decreased expression of certain genes encoding proteins involved in glycolysis and gluconeogenesis (for example, *PCK1*, *ALDOB*), which ultimately would also decrease lipogenesis (Fig. 4d). Hints of adverse effects were however also noted for C2. We observed various DEGs suggestive of induction of epithelial-to-mesenchymal transition through TGFβ-mediated extracellular matrix remodeling (Fig. 4e and Supplementary Fig. 12c), accompanied by reduced expression of specific hepatocyte and epithelial markers (Supplementary Fig. 12d). These findings could well explain the presence of *FGF19* gene amplifications in liver cancer^49^.

Collectively, either direct (ACCi, FASi, DGAT2i) or indirect (FXRa, hFGF19) repression of DNL appeared to represent a unified and highly effective mechanism through which steatosis can be resolved, while cellular ‘side effects’ are largely treatment cluster dependent (Fig. 4f).

### Development of FatTracer as a genetic steatosis recorder

**Fig. 5:**
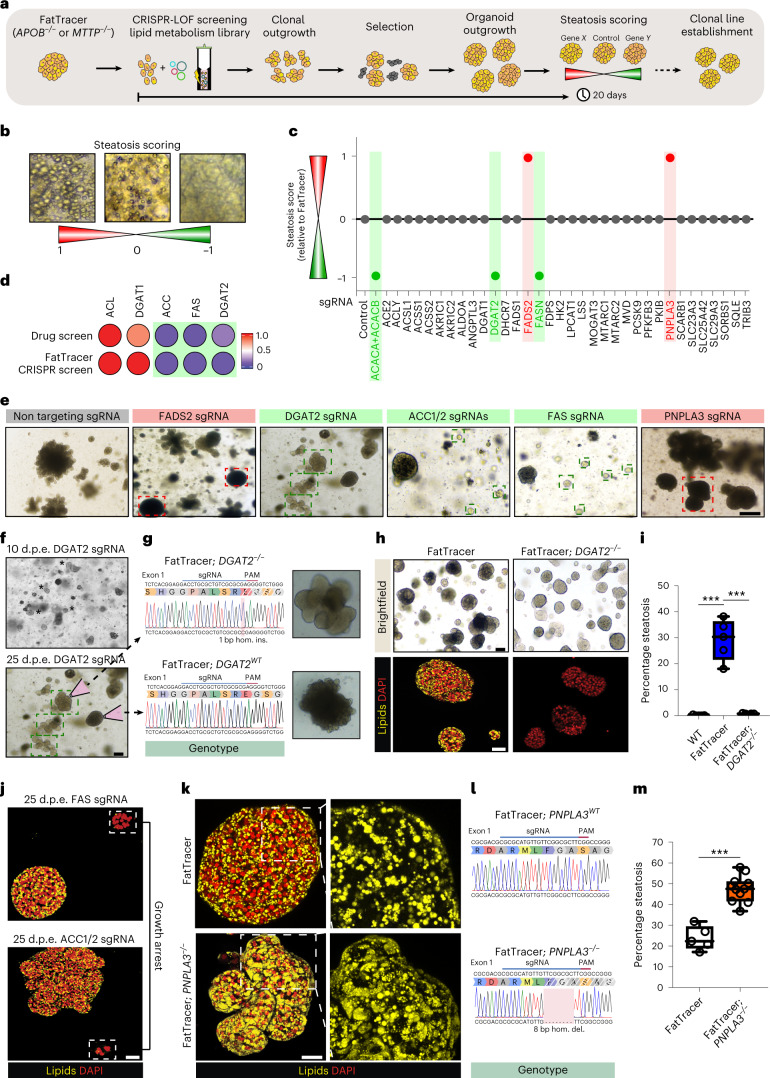
Development of FatTracer as a CRISPR screening platform identifies steatosis mediators. **a**, Strategy used to perform CRISPR-LOF screening in FatTracer. **b**, Brightfield images exemplifying the steatosis scoring scale, where 0 (white) reflects the baseline steatosis level of FatTracer and 1 (red) and −1 (green) indicate increased and decreased steatosis, respectively. **c**, CRISPR-LOF screening results of 35 candidates evaluated in FatTracer using the steatosis scoring scale. **d**, Comparative analysis of results from FatTracer CRISPR-LOF screening and drug screening for the indicated gene targets. The lipid scoring system was used to quantify steatosis levels (Fig. 3c). Green boxes highlight steatosis-reducing effects. **e**, Brightfield images of outgrowing FatTracer organoids following CRISPR targeting of the indicated genes. Green boxes highlight lighter (fat-free) organoids, red boxes highlight darker (more fatty) organoids. **f**, Brightfield images of outgrowing FatTracer organoids following CRISPR targeting of *DGAT2* 10 and 25 days post electroporation (d.p.e.). Asterisks indicate lighter (less lipid-containing) organoids. **g**, Sanger trace sequences of the picked organoid lines highlighted in **f**. **h**, Brightfield images and Nile Red lipid staining of FatTracer and FatTracer; *DGAT2*^*−/−*^ organoids. **i**, Quantification of the percentage of steatosis in FatTracer, FatTracer; *DGAT2*^*−/−*^ and WT organoids (all from the same donor). *n* = 5 independent replicates per genotype. Two-tailed *t*-test, ***P < 0.0001. **j**, Nile Red lipid staining of outgrowing FatTracer organoids following CRISPR targeting of *FASN* or *ACACA* + *ACACB* 25 d.p.e. **k**, Nile Red lipid staining of FatTracer and FatTracer; *PNPLA3*^*−/−*^ organoid lines. **l**, Sanger trace sequences of *PNPLA3* genotypes of the organoid cultures shown in **k**. **m**, Quantification of the steatosis level in FatTracer and FatTracer; *PNPLA3*^*−/−*^ organoids. *n* = 11 independent replicates from three FatTracer; *PNPLA3*^*−/−*^ clonal lines; *n* = 5 independent replicates from the parental FatTracer line. Two-tailed *t*-test, ***P < 0.0001. **i**,**m**, The box indicates the 25–75th percentiles, the center line indicates the median and the whiskers indicate minimum and maximum values. **e**,**f**,**h**,**j**,**k**, Representative of *n* = 3, 6, 6, 3 and 4 independent experiments, respectively, using both *APOB*^*−/−*^ and *MTTP*^*−/−*^ organoids from two donors as FatTracer. Scale bars, 100 μm (**e**,**f**), 200 μm (brightfield) and 50 μm (fluorescence) (**h**), 50 μm (**j**) and 25 μm (**k**).

We next questioned whether we could extend the use of our organoid models to identify steatosis mediators and putative targets. In that regard, *APOB*^*−/−*^ and *MTTP*^*−/−*^ organoids possess unique features as steatosis models, including (1) their high similarity in drug response to FFA-induced steatosis organoids; (2) their long-term expansion as ‘naturally’ steatotic hepatocytes, allowing further genomic modification; and (3) their considerable steatosis levels that allow easy identification of aggravating or alleviating effects following drug or gene perturbation. We therefore exploited these features to leverage our mutants as a human platform for CRISPR-based genetic screening of potential steatosis modulators and possible drug targets. We named this platform FatTracer (Fig. 5a). Inspired by the very specific target dependency that emerged from the drug screening, we compiled a CRISPR–Cas9-based loss-of-function (LOF) library of lipid metabolism-related genes (*n* = 35 genes). This library included a broad variety of genes encoding, amongst others, lipid metabolism enzymes, transporters and indirect mediators, for evaluation in FatTracer (Supplementary Table 3). To validate the use of FatTracer for CRISPR-LOF screening, we included some effective targets identified in our drug screening (that is, DGAT2, ACC (*ACACA/B*) and FAS (*FASN*)), as well as PNPLA3 as a proxy to interrogate NAFLD risk genes. We transfected single cells with plasmids encoding for Cas9 and the pertinent sgRNA, as well as plasmids for antibiotic resistance selection to enrich for transfected cells. Every gene was targeted independently. When the outgrowing clonal organoids had fully grown, we analyzed the phenotypes in comparison with a control condition in which organoids were transfected with a nontargeting sgRNA (Fig. 5a,b). This approach allowed us to evaluate the effects of gene KO in hepatic steatosis within a timeframe of only ±20 days (Fig. 5a).

We first focused on the validation genes (Fig. 5c–m and Supplementary Fig. 13a). Transfection with Cas9-DGAT2 sgRNA resulted in the appearance of lighter (less lipid-containing) organoids, while other organoids still presented the typical steatosis phenotype (Fig. 5e,f). We analyzed the genotypes of both these still-steatotic and fat-resolved outgrowing organoids. Across multiple independent experiments, and using both *APOB*^*−/−*^ and *MTTP*^*−/−*^ organoids as FatTracer models, we found 100% fidelity in genotype–phenotype correlation—that is, fat-resolved organoids always carried homozygous *DGAT2* mutations while steatosis-presenting organoids carried no or heterozygous *DGAT2* mutations (Fig. 5g). Nile Red staining and quantitative assessments corroborated the strong reduction in the steatosis level upon *DGAT2* KO, reverting to WT organoid levels (Fig. 5h,i). Of note, clonal FatTracer; *DGAT2*^*−/−*^ lines were viable and proliferative. Under conditions targeting either *FASN* (encoding FAS) or *ACACA* + *ACACB* (encoding ACC1/2), we consistently noted outgrowth of fat-free organoids that remained very small (Fig. 5e,j and Supplementary Fig. 13a). These organoids quickly ceased to grow, and therefore organoid lines could not be derived. These CRISPR experiments were in full agreement with our findings from the drug screening and treatment-based clustering analysis (Fig. 5d), which suggested inhibition of proliferation following ACC or FAS inhibition. This highlights FatTracer’s potential as a predictive platform for evaluation of gene effects on both steatosis levels and cellular phenotypes, such as proliferation.

We then focused our attention on the other evaluated lipid metabolism-related genes (Fig. 5c). The majority of targeted genes did not induce phenotypic differences in FatTracer (Supplementary Fig. 13a). This included, for example, *DGAT1*, again in agreement with our previous drug screenings, further highlighting high target specificity (Fig. 5c,d). We noted obvious phenotypic changes to FatTracer following KO of *PNPLA3* (Fig. 5c,e,k and Supplementary Fig. 13a). Clonal FatTracer; *PNPLA3*^*−/−*^ lines displayed a substantially worsened steatosis level, reaching around 50%, which is near double the steatosis level of FatTracer (Fig. 5k–m). These findings aligned well with our findings on steatosis induction following *PNPLA3* KO in WT organoids (Fig. 1d–f), and suggested FatTracer’s potential in evaluation of NAFLD risk genes.

### FatTracer identifies FADS2 as a critical steatosis modulator

**Fig. 6:**
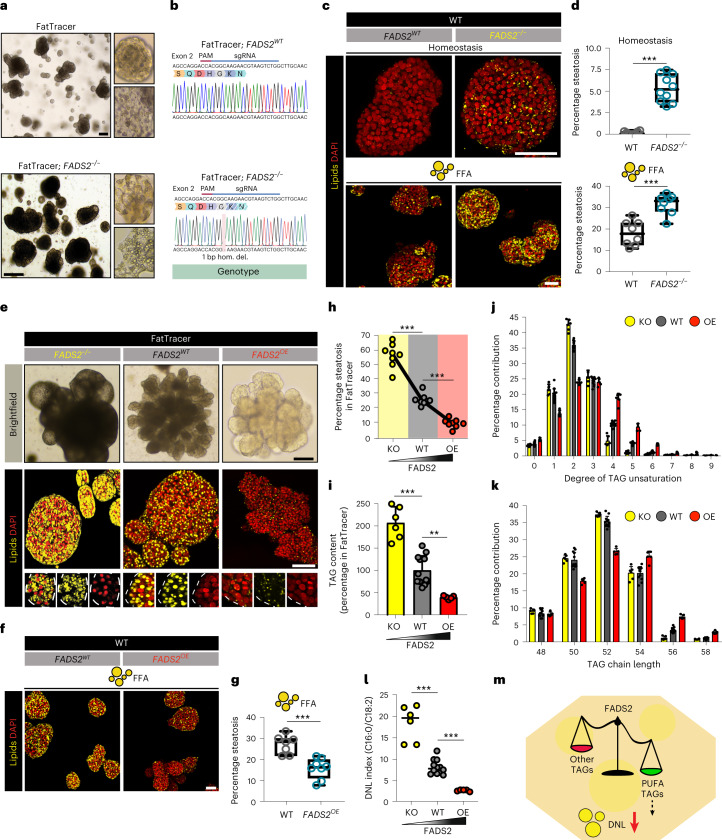
FADS2 is a key modulator of steatosis levels in human hepatocytes. **a**, Brightfield images of FatTracer and FatTracer; *FADS2*^*−/−*^ organoids. **b**, Sanger trace sequences of *FADS2* genotypes of the organoid cultures shown in **a**. **c**, Nile Red lipid staining of *FADS2*^*WT*^ and *FADS2*^*−/−*^ organoids in homeostasis and following FFA exposure (320 μM). **d**, Quantification of the percentage of steatosis in homeostasis (top) and after FFA exposure (320 μM) (bottom) in *FADS2*^*WT*^ and *FADS2*^*−/−*^ organoids. *n* = 10 (homeostasis) and *n* = 8 (FFA) independent replicates from two clonal lines per genotype from two donors. Two-tailed nested *t*-test: homeostasis, ***P < 0.0001; FFA, ***P = 0.0001. **e**, Brightfield images and Nile Red lipid staining of FADS2 variant FatTracer organoids. **f**, Nile Red lipid staining of *FADS2*^*WT*^ and *FADS2*^*OE*^ organoids after FFA exposure (500 μM). **g**, Quantification of the percentage of steatosis after FFA exposure (500 μM) in *FADS2*^*WT*^ and *FADS2*^*OE*^ organoids. *n* = 7 independent replicates for *FADS2*^*WT*^, *n* = 8 independent replicates for *FADS2*^*OE*^ from two clonal lines per genotype from two donors. Two-tailed nested *t*-test: ***P = 0.0004. **h**, Quantification of the percentage of steatosis in FADS2 variant FatTracer organoids. *n* = 8 independent replicates from two different clonal lines per genotype from two donors. Two-tailed nested *t-*test: ***P < 0.0001. **i**, Quantification of TAG content in FatTracer following *FADS2*^*−/−*^ and *FADS2*^*OE*^ relative to *FADS2*^*WT*^ (mean ± s.d.). *n* = 6 independent measurements in three clonal lines from two donors for FatTracer; *FADS2*^*−/−*^ and FatTracer; *FADS2*^*OE*^; *n* = 12 independent measurements in FatTracer; *FADS2*^*WT*^ from the same two donors. Two-tailed *t-*test: KO versus WT, ***P < 0.0001; OE versus WT, **P = 0.0011. **j**,**k**, Relative distribution of degree of TAG unsaturation (**j**) and chain length (**k**) in FADS2 variant FatTracer organoids (mean ± s.d.). **l**, Quantification of DNL index (C16:0/C18:2 ratio) based on the fatty acid composition of the TAG in FADS2 variant FatTracer organoids. Two-tailed *t-*test, ***P < 0.0001. **j**–**l**, Sample sizes as in **i**. **m**, Proposed role of FADS2 in regulation of hepatic steatosis. **d**,**g**, The box indicates the 25–75th percentiles, the center line indicates the median and the whiskers indicate minimum and maximum values. **a**,**c**,**e**,**f**, Representative of *n* = 4, 3, 4 and 3 independent experiments, respectively. Scale bars, 200 μm (**a**), 50 μm (**c**,**e**,**f**).

From this CRISPR screen, FADS2 (but not FADS1) emerged as a critical player in steatosis (Fig. 5c,e and Supplementary Fig. 13a). FADS2 is a delta-6 desaturase mediating the rate-limiting step in the biosynthesis of polyunsaturated fatty acids (PUFAs). A causative role of FADS2 in NAFLD has not previously been implied, nor has FADS2 been investigated as a potential target. Clonal FatTracer; *FADS2*^*−/−*^ lines presented as much darker (more lipid-containing) organoids compared with FatTracer (Fig. 6a). We confirmed their genotypes (Fig. 6b), and immunofluorescence staining corroborated the loss of FADS2 protein (Supplementary Fig. 14a). To further evaluate the role of FADS2, we questioned whether *FADS2* KO would predispose WT human hepatocytes to steatosis. We therefore generated *FADS2*^*−/−*^ organoid lines, which indeed spontaneously accumulated lipids in basal culture conditions (roughly 5% steatosis) (Fig. 6c,d). We next used these lines to elucidate the interplay between the lack of FADS2 and a FFA-induced steatosis trigger. When challenged with a mild dose of FFAs (320 μM), *FADS2*^*−/−*^ organoids displayed a markedly aggravated steatosis phenotype compared with *FADS2*^*WT*^ organoids challenged with the same FFA dose (Fig. 6c,d), further strengthening the evidence of an important role for FADS2 in balancing lipid homeostasis.

### FADS2 overexpression protects against steatosis

Prompted by these findings, we questioned whether overexpression of FADS2 would be beneficial for the steatosis phenotype in terms of (1) resolving existing steatosis and/or (2) being protective against its development. We employed a transposase-based integration of a CAG-FADS2 cassette to drive stable and constitutive overexpression of FADS2 (*FADS2*^*OE*^) in transfected cells (Supplementary Fig. 14b). We first overexpressed FADS2 in FatTracer. Notably, we observed the appearance of lighter (less lipid-containing) outgrowing organoids (Supplementary Fig. 14c,d). These findings were also corroborated by fluorescent activated cell sorter analysis of the outgrowing organoids with a fluorescent lipid dye (Supplementary Fig. 14e). We derived multiple clonal FatTracer; *FADS2*^*OE*^ lines (ranging from around two–25-fold overexpression (Supplementary Fig. 14f,g)) and found that FADS2 overexpression (further confirmed by antibody staining (Supplementary Fig. 14a)) led to almost complete resolution of steatosis (roughly 70% reduction) (Fig. 6e,h and Supplementary Fig. 14h), without altering the organoids’ growth capacity and proliferation (Supplementary Fig. 14i). Of note, only a mild threshold level of FADS2 overexpression was needed to reduce steatosis (Supplementary Fig. 14j,k). We next generated *FADS2*^*OE*^ lines in WT organoids to evaluate whether FADS2 overexpression could confer protection against FFA-induced steatosis. We applied the same dosage of FFAs as used for previous drug screenings (500 μM) and found that FADS2 overexpression markedly reduced the extent of lipid accumulation (Fig. 6f,g). Therefore, modulation of the level of FADS2 expression directly determines the level of steatosis within hepatocytes (Fig. 6e,h), both in developing and pre-existing steatosis.

### Mechanisms of FADS2 protection in steatosis

Given the role of FADS2 in fatty acid synthesis, we searched for mechanistic cues by performing lipidomics on FatTracer organoids in baseline (*FADS2*^*WT*^) and following *FADS2*^*−/−*^ and *FADS2*^*OE*^. We first analyzed total TAG content within these different FADS2 variant organoids, which photocopied the observed steatosis trend (Fig. 6i). Relative to FatTracer; *FADS2*^*WT*^, FatTracer; *FADS2*^*−/−*^ organoids had an increase of around 200% in TAG content whereas *FADS2*^*OE*^ organoids had a marked reduction of >50% (Fig. 6i). The vast majority of TAG species were reduced following *FADS2*^*OE*^ and conversely increased with *FADS2*^*−/−*^ (Supplementary Fig. 15a,b). We then evaluated TAG characteristics (Supplementary Fig. 15a–f). In line with its desaturase activity, TAG unsaturation levels were notably changed. FatTracer; *FADS2*^*OE*^ organoids were enriched with TAG containing fatty acid chains with a higher degree of unsaturation, while conversely FatTracer; *FADS2*^*−/−*^ organoids displayed an increase in TAG with lower unsaturation levels (that is, containing at least one saturated fatty acid) (Fig. 6j).

TAG chain length analysis revealed a different trend. FatTracer; *FADS2*^*−/−*^ organoids displayed near-identical chain length profiles as compared with FatTracer; *FADS2*^*WT*^, indicating that loss of FADS2 does not impair the overall generation of longer TAG species (Fig. 6k). The aggravated steatosis under loss of FADS2 is therefore solely associated with increased abundancy in TAG species containing fatty acid chains with lower degrees of unsaturation. Instead, FatTracer; *FADS2*^*OE*^ organoids were enriched for longer-chain TAG species (54 C, 56 C, 58 C) at the expense of shorter TAG species (50 C, 52 C) (Fig. 6k). Shorter-chain TAG species represent those consisting of newly synthesized fatty acids^50^. A shift towards incorporation of longer-chain ‘old’ PUFAs following FADS2 overexpression therefore suggested decreased endogenous fatty acid production. To further test this hypothesis, we calculated the DNL index (C16:0/C18:2)^51^ in TAG of the FADS2 variant FatTracer organoids. As compared with FatTracer; *FADS2*^*WT*^ organoids, the DNL index was sharply increased following FADS2 loss while it decreased in FADS2-overexpressing organoids (Fig. 6l). Thus, a FADS2-regulated PUFA–DNL axis may represent a functional mechanism to influence steatosis levels within the hepatocyte (Fig. 6m).

## Discussion

In this study we developed human organoid models of hepatic steatosis. Organoid lines (unlike cell lines) can be directly generated from healthy WT cells from donors of different genetic backgrounds^23,24^. The ability to reproduce findings within the same organoid line allows for high reproducibility. An important and exclusive feature (contrasting with other primary or pluripotent stem cell-derived hepatocyte systems) is their proliferative nature, enabling the generation of large organoid numbers in a relatively short timeframe. This allows application of robust and versatile genome-engineering protocols of WT hepatocytes^24–26^. With precise gene editing, we show that the effects of gene mutations or SNPs (as exemplified for PNPLA3 I148M) on steatosis risk can be addressed with high phenotypic resolution. Altogether, these features allowed the development of scalable (personalized) organoid platforms paired with a drug–CRISPR toolkit for NAFLD target identification and testing. The developed organoid models capture different and clinically relevant biological mechanisms of steatosis: while the FFA model captures primarily steatosis driven by a surplus of FFAs (approximating a Western diet), *APOB*^*−/−*^ and *MTTP*^*−/−*^ organoids develop steatosis through accumulation of lipids derived from DNL.

Through drug screening conjugated to in-depth mechanistic analysis, we discovered that repression of DNL (either directly or indirectly) is a common and highly effective mechanism to reduce steatosis, even under FFA-induced steatosis where DNL is not the primary trigger (but instead exogenously provided palmitic acid and oleic acid). Other possible approaches, such as increasing fatty acid oxidation, do not appear to be as beneficial, as exemplified by the observed lack of effects of PPAR agonists. Clinical studies have indeed reported increased rates of DNL as a distinct feature of NAFLD patients^52^, making it a potential, clinically attractive mechanism to combat steatosis. The drug treatment-based clustering approach allowed classification of effective drugs based on specific molecular signatures. These signatures helped not only to decipher the common mechanisms of different drug actions (for example, that a FXR–FGF19 signaling axis effectively reduces DNL in hepatocytes), but also to predict possible adverse hepatocyte effects (for example, impaired liver regeneration capacity following treatment with ACC and FAS inhibitors). While organoid responses across the different steatosis models were generally conserved, our data hinted at the possibility that genetic risk may influence drug treatment outcomes. Carrying the PNPLA3 I148M variant attenuated organoid responses towards steatosis-reducing drugs. This was particularly evident for the FXR–FGF19 drug axis; a mechanistic explanation for this remains to be further investigated. Whether personalized medicine could be of value for NAFLD is currently unexplored^53^, but our organoid data hint at such possibility.

In the hunt for drug targets, we exploited *APOB*- and *MTTP*-mutant organoids as a real-time genetic steatosis recorder (FatTracer) for CRISPR-LOF screening. This approach allows rapid functional investigation of candidate genes in a human context and in a high-throughput manner, requiring small starting material, with high reproducibility. Targets that emerged from the drug screening were also identified in this genetic screening. Moreover, LOF screening predicted the functional importance of PNPLA3. In addition to the genes screened in the current study, FatTracer in principle offers the possibility of performing (pooled) CRISPR screens at a genome-wide level. This could include evaluation of the role of many (often functionally unconfirmed) GWAS-associated NAFLD risk variants in the context of steatosis and under different culture conditions—for example, by evaluation of different carbohydrate sources and concentrations as well as insulin levels. The development of the PLIN2 lipid reporter lines allows the combination of steatosis organoid models with automated imaging systems for higher-throughput drug and CRISPR screenings.

This platform has some inherent limitations. Because FatTracer is a model in which VLDL secretion is defective, care should be taken when interpreting findings regarding the function of genes encoding proteins involved in lipid export. The same holds true for genes encoding proteins interacting at the level of organ crosstalk (for example, as is the case for PCSK9 screened here, which acts on circulating LDL particles). As a purely hepatocyte system, gene effects in other liver cell types or with roles in multicellular crosstalk cannot be studied in the current version of the organoid platform. FatTracer evaluates gene–steatosis relationships in a pre-existing steatosis state with the capacity to highlight both reducing and aggravating effects. To capture also the transition from a healthy to diseased state, evaluation with WT organoids can be performed in parallel.

The promise of FatTracer is underscored by our findings on FADS2. Mouse studies yielded controversial results on the role of FADS2 in the context of fatty liver^54–56^. Initially identified from our CRISPR-LOF screening by exacerbated steatosis, we found that increasing FADS2 protein could effectively prevent and resolve steatosis. Mechanistically this was linked to a reduced total TAG content, with a concomitant favorable internal shift in TAG composition where FADS2 activity determines PUFA abundancy. Saturated fats are more harmful and potent at promoting the development of fatty liver as compared with PUFAs^57^. Livers of NAFLD patients display reduced PUFA content^58^, while promotion of PUFA intake can reduce liver fat content in obese patients^59^. How PUFAs may exert beneficial effects on steatosis is unclear; our lipidomic findings hint at repression of DNL. The *FADS* gene cluster is SNP rich, with various associations with lipid-related traits including plasma lipid levels, glucose homeostasis, type 2 diabetes risk, dyslipidemia and metabolic syndrome^60,61^. Of particular relevance, a recent study on novel NAFLD risk loci reported on a potential SNP in *FADS2* (ref. ^62^). Our functional data position FADS2 as an important steatosis mediator and putative target, and furthermore suggest that genetic variation in *FADS2* may confer susceptibility to steatosis.

In this study we have focused on steatosis, which at the hepatic level is primarily a hepatocyte disease. At more advanced clinical stages, such as NASH, the liver presents with inflammation and fibrosis through involvement of other liver cell types. Nonetheless, resolution of steatosis is often associated with downstream improvements of NASH^63^, and being able to halt progression from steatosis to NASH is of key interest^16^. Implementation of cocultures with nonparenchymal cells (for example, Kupffer and stellate cells), similar to multicellular liver spheroids/organoids^20,64^, may extend the application of our organoid models and CRISPR and drug-screening approaches to later stages of the NAFLD–NASH spectrum.

## Methods

### Organoid culture

The use of human fetal livers for research was approved by the Dutch Ethical Medical Council (Leiden University Medical Center). Human fetal hepatocyte organoid lines were established and cultured in HEP medium as described by Hu et al.^23^ and Hendriks et al.^24^. In brief, hepatocytes were isolated by collagenase type IV (Sigma-Aldrich) digestion and enriched for by using low-speed centrifugation. Hepatocytes were washed with AdvDMEM+++ (AdDMEM/F-12 medium supplemented with 1× GlutaMAX, 10 mM HEPES and 100 U ml^–1^ penicillin/streptomycin solution (all Thermo Fisher)) and seeded in 100 μl of BME (Cultrex) suspension (2:1 BME:AdvDMEM+++) per well of a 12-well plate (three droplets per well (33 μl per droplet)). Organoids were maintained in HEP medium (AdvDMEM+++ supplemented with 15% RSPO1-conditioned medium (in-house production), 1× B-27 Supplement Minus Vitamin A (Thermo Fisher), 2.5 mM nicotinamide (Sigma-Aldrich), 1.25 mM *N*-acetyl-l-cysteine (Sigma-Aldrich), 50 ng ml^–1^ hEGF, 50 ng ml^–1^ hFGF7, 50 ng ml^–1^ hFGF10, 50 ng ml^–1^ hHGF, 20 ng ml^–1^ hTGFα (all Peprotech), 10 nM gastrin (Sigma-Aldrich), 3 μM CHIR-99021 (Sigma-Aldrich), 1 μM A 83-01 (Tocris), 5 μM Y-27632 (AbMole) and 50 μg ml^–1^ primocin (InvivoGen)). Organoids were typically passaged by mild mechanical shearing with a P1000 pipette every 7–10 days at a ratio of 1:2–1:4. During the first few days of organoid line establishment, HEP medium was supplemented with extra Y-27632 (final concentration, 10 μM) to minimize anoikis.

### Medium withdrawal experiments

To probe the effect of selected medium components on the steatosis phenotype, HEP medium without RSPO1-conditioned medium and/or B-27 Supplement Minus Vitamin A was prepared. Alternatively, rather than using AdvDMEM+++ as a base medium, HEP medium was prepared using William’s Medium E+++ (William’s Medium E without phenol red (Thermo Fisher)) supplemented with 1× GlutaMAX, 10 mM HEPES and 100 U ml^–1^ penicillin/streptomycin solution). Organoids were passaged and plated in new BME droplets, and subsequently cultured in these different culture media and collected for downstream analyses after 2 weeks of culture (including one splitting event).

### Steatosis induction by exogenous FFA loading

Organoids were made steatotic by supplementing the culture medium with a concentrated mixture of exogenous FFAs (oleic acid and palmitic acid; both Sigma-Aldrich) in an equal ratio. FFAs were dissolved in 100% ethanol (200 mM stocks) and conjugated to bovine serum albumin (BSA, Sigma-Aldrich) using 10% BSA-PBS to stock concentrations of 8 mM. Vehicle stocks were prepared in an identical manner using 100% ethanol. Before FFA loading, organoids were plated into smaller BME droplets (15 μl per droplet) to facilitate FFA penetration.

### CRISPR gene KO

Organoids were CRISPR engineered using Cas9/nonhomologous end-joining-mediated gene disruption. Single-guide RNAs were designed using an online web-tool (www.atum.bio/eCommerce/cas9/input) and cloned into the pSPgRNA plasmid (Addgene, no. 47108) as described by Ran et al.^65^. The sgRNA sequences of targeted genes are given in Supplementary Table 3. To provide Cas9, we used a plasmid expressing SpCas9 as well as mCherry for visualization of transfected cells (Addgene, no. 66940). To enrich for transfected cells we made use of an in-house, two-plasmid transposon system comprising a *piggyBac* transposase and a donor plasmid with inverted terminal repeats flanking a hygromycin resistance gene cassette. Human fetal hepatocyte organoids were transfected by electroporation, as described previously^24^. Briefly, organoids were converted to single cells using Accutase (Thermo Fisher), washed twice with AdvDMEM+++ and resuspended in 130 μl of Opti-MEM (Thermo Fisher) containing the DNA mixture. Typically, one to two wells of a 12-well plate and a maximum 20 μg of DNA were used per electroporation. Electroporation was performed in Nepa electroporation cuvettes (2-mm gap) with a NEPA21 electroporator (both Nepagene) using the settings described in Hendriks et al.^24^. After 20-min recovery in HEP medium with additional Y-27632 (final concentration, 10 μM), single cells were recovered, washed with AdvDMEM+++ and plated in 100 μl of BME suspension into one well of a 12-well plate (three droplets per well). Cells were cultured in complete HEP medium with additional Y-27632 (final concentration, 10 μM) until small organoids appeared, after which these were shifted to regular HEP medium. When organoids were cotransfected with the hygromycin–*piggyBac* two-plasmid system, selection was started when organoids of small size had formed (typically after 7 days). Hygromycin B Gold (100 μg ml^–1^, InvivoGen) was kept until selection was complete (roughly 7–12 days). Single surviving organoids were picked, converted to small fragments/single cells by Accutase, plated into a single BME droplet well of a 24-well plate and expanded into clonal lines. When no selection strategy was used, organoids were picked based on their fat phenotypes and grown into clonal lines as described above.

### CRISPR prime editing

The PNPLA3 I148M mutation was introduced by PE3 prime editing^33^. Prime editing guide RNAs and PE3 sgRNA were designed using pegFinder^66^. The pegRNAs were generated as described by Anzalone et al.^33^ and the PE3 sgRNA was cloned as described above. Spacer and 3’ extensions for pegRNAs and the PE3 sgRNA are given in Supplementary Table 3. In sum, the transfection mixture consisted of the specific pegRNA plasmid containing the sgRNA and desired edit, the common PE3 sgRNA plasmid to induce the second nick and the PE2 plasmid (Addgene, no. 132775), as well as the hygromycin–*piggyBac* two-plasmid system to facilitate selection. Outgrowing hygromycin-resistant clones were picked and genotyped, and clonal lines were established as described above.

### FADS2 overexpression

To overexpress human FADS2 we generated a transposable construct for use in conjunction with the *piggyBac* transposase. Total RNA was extracted from human hepatocyte organoids and used as template for complementary DNA synthesis using the SuperScript IV kit (Thermo Fisher) and FADS2 was PCR amplified and gel purified. Using PBCAG-eGFP (Addgene, no. 40973) as a backbone, we replaced the eGFP sequence with FADS2 using In-Fusion cloning (Takara Bio) to generate the CAG-FADS2 plasmid. The CAG-FADS2-P2A-tdTomato plasmid was generated in a similar manner, using P2A-tdTomato as an additional cloning insert. FADS2 overexpression was confirmed by quantitative PCR with reverse transcription analysis using iQSYBRGreen mix (Bio-Rad) with the quantitative PCR primers FADS2_fw: 5′ GACCACGGCAAGAACTCAAAG 3′ and FADS2_rev: 5′ GAGGGTAGGAATCCAGCCATT 3′.

### Genotyping of organoid lines

To characterize organoid lines for their NAFLD risk factor genotypes, DNA was extracted from each line using the Quick-DNA Microprep kit (Zymo). PCR reactions were performed to amplify the genomic region encompassing the genomic region of interest and PCR products were Sanger sequenced. To genotype the various mutant organoid lines, as soon as sufficient organoid material was present DNA was extracted and PCR reactions performed using primers encompassing the sgRNA/Cas9-targeted area. PCR products were subsequently Sanger sequenced and, when necessary, genotypes were deconvoluted using the ICE v.2 CRISPR tool and PCR products were further subcloned to discriminate between alleles whenever appropriate.

### Drug screening

Compound stocks of all drugs were made in DMSO (Sigma-Aldrich), except for recombinant hFGF19 and hFGF21 which were reconstituted in AdvDMEM+++. Drugs were dissolved in HEP medium with a maximum final concentration of 0.2% DMSO. Details on the drug panel, their targets and concentrations tested are described in Supplementary Table 2. *APOB*^*−/−*^ and *MTTP*^*−/−*^ organoids were exposed to drugs or vehicle in 24-well plates for 7 days with two medium changes. WT organoids (*PNPLA3*^*WT*^ or *PNPLA3*^*I148M/I148M*^, engineered from the same donor) were first made steatotic by preincubation with 500 μM FFA (oleic acid and palmitic acid, 1:1 ratio) for 3 days. Then, organoids were treated with the different drugs, still in the presence of 500 μM FFA, for 7 days with two medium changes. All organoids were harvested for subsequent lipid staining and lipid scoring as described below. Drug effects were evaluated by assessment of lipid droplet fluorescence characteristics of all organoids within the whole well. Quantitative analyses were performed in representative organoid cultures (*n* ≥ 3) per drug concentration per steatosis organoid model. Drug effects were validated in at least *n* = 2 independent experiments.

### CRISPR screening

*APOB*^*−/−*^ or *MTTP*^*−/−*^ organoid lines (FatTracer), generated without previous hygromycin resistance, were used for CRISPR screening. Organoids were transfected with the sgRNA plasmid targeting the gene of interest, a Cas9-expressing plasmid and the hygromycin–*piggyBac* two-plasmid system in a manner identical to that described above. For each targeted gene, organoids from one well of a 12-well plate were used as starting material. The sgRNA sequences of the screened genes are given in Supplementary Table 3. Outgrowing hygromycin-resistant organoids were carefully inspected by brightfield microscopy and compared with control surviving organoids (mock-transfected organoids with Cas9 and a nonhuman sgRNA), using a binary classification of lighter (–1), similar (0) or darker (1). To ensure that the effects of the targeted genes were not evaluated during the energy-demanding phase of clonal outgrowth, we scored the phenotype of the organoids after they reached a steady-state phase—that is, when the organoids were of a size suitably large for establishment of a clonal line. Upon indication of different phenotypes between surviving organoids (that is, impacting on the steatosis phenotype), organoids were picked, grown into clonal lines and genotyped as described above. Alternatively, organoids were directly processed for lipid staining when clonal line establishment was not possible. CRISPR experiments were repeated at least in *n* = 2 independent experiments using both *APOB*^*−/−*^ and *MTTP*^*−/−*^ lines from two donors as FatTracer. Notable findings were followed up in *n* ≥ 3 independent experiments using multiple clonal lines from two donors.

### Lipid and immunofluorescence staining

Organoids were carefully harvested in cold AdvDMEM+++ and washed once with cold AdvDMEM+++. Sedimented organoids were fixed in 4% formaldehyde at room temperature (RT) for 30–60 min. For lipid staining, organoids were washed twice with PBS and incubated with Nile Red (0.5 μg ml^–1^) and DAPI (1 μg ml^–1^) (both Thermo Fisher) for 20 min at RT. Organoids were washed twice with PBS and transferred in 100 μl of PBS to one well of a 96-well black SensoPlate (Greiner Bio-One) for imaging analysis. For immunofluorescence staining, fixed organoids were first washed twice with PBS and then simultaneously blocked and permeabilized using 5% BSA and 0.3% Triton-X in PBS at RT for 1 h. Organoids were washed once with 0.5% BSA-PBS and subsequently incubated with primary antibodies in 2.5% BSA-PBS overnight at 4 °C. The primary antibodies used were: mouse anti-ApoB (989529), no. MAB41241-SP, Novus Biologicals, dilution 1:50; rabbit anti-beta-catenin (H-102), no. sc-7199, Santa Cruz, dilution 1:200; rabbit anti-FADS2/Delta-6 Desaturase (aa79-108), no. LS-C165916, LSBio, dilution 1:50; rat anti-Ki-67 (SolA15), no. 14-5698-82, Thermo Fisher, dilution 1:1,000; rabbit anti-MTP, no. ab63467, Abcam, dilution 1:500; and sheep anti-PNPLA3/Adiponutrin, no. AF5208, R&D systems, dilution 1:100. After three washes with 0.5% BSA-PBS, organoids were incubated with appropriate Alexa Fluor secondary antibodies (all Thermo Fisher, at dilution 1:1,000) in 2.5% BSA-PBS for 2–4 h at RT. The secondary antibodies used were: Alexa Fluor 488 anti-mouse, A11029; Alexa Fluor 488 anti-rabbit, no. A21206; Alexa Fluor 568 anti-sheep, no. A21099; Alexa Fluor 568 anti-rabbit; Alexa Fluor 647 anti-rat, no. A21247; and Alexa Fluor 647 anti-rabbit, no. A21245. Cell membranes were stained with Phalloidin-Atto 647 N (no. 65906, Sigma-Aldrich, dilution 1:2,000). Organoids were washed once with 0.5% BSA-PBS, after which they were incubated with DAPI (1 μg ml^–1^) in 0.5% BSA-PBS for 20 min at RT and washed once more with 0.5% BSA-PBS. Organoids were then transferred in 100 μl 0.5% BSA-PBS to one well of a 96-well black SensoPlate.

### Confocal imaging and lipid scoring

Stained organoids were imaged on a Leica Sp8 confocal using Leica LAS X software (v.1.1). Fluorescent images were processed using either Photoshop CS4 or Fiji (v.2.0.0) software. Lipid scores were calculated using Fiji. The lipid score is defined by integrating lipid droplet fluorescence and lipid droplet area coverage as follows: a fluorescence threshold derived from the lipid droplet signal is determined on Z-projected images to convert them into binary images. Note that we did not observe notable differences between measurements of steatosis levels following assessment in three versus two dimensions. The region of interest (organoid surface area) is defined based on fluorescence signal occupancy from counterstained DAPI^+^ nuclei. Then, particle measurement analysis is performed to define the fluorescence area covered within the defined region of interest. The lipid score represents a normalized score of the resulting data on a linear 0–1 scale, where the average calculated lipid droplet area coverage values from WT organoids are arbitrarily set to 0 while those from either vehicle-treated *APOB*^*−/−*^ or *MTTP*^*−/−*^ organoids or vehicle-treated FFA-exposed WT organoids are set to 1, allowing scoring of drug effectiveness within these boundaries. See also Supplementary Fig. 7b for a visual explanation. Lipid droplet area coverage (percentage steatosis) is determined in an identical manner to the lipid score but without the final normalization steps.

### Transmission electron microscopy

Organoids were fixed with 1.5% glutaraldehyde in 0.1 M cacodylate buffer at 4 °C for 24 h. Then, organoids were washed with 0.1 M cacodylate buffer and postfixed with 1% osmium tetroxide in the same buffer containing 1.5% potassium ferricyanide in the dark at 4 °C for 1 h. Samples were dehydrated in ethanol, infiltrated with Epon resin for 2 days, embedded in the same resin and polymerized at 60 °C for 48 h. Ultrathin sections were cut using a Leica Ultracut UCT ultramicrotome (Leica Microsystems) and mounted on Formvar-coated copper grids. Sections were stained with 2% uranyl acetate in 50% ethanol and lead citrate. Sections were observed under a Tecnai T12 Electron Microscope equipped with an Eagle 4k × 4k CCD camera (Thermo Fisher). To specifically preserve and visualize lipid droplet morphology, organoids (fixed similarly to that described above) were instead high-pressure frozen using a Leica HPF. Freeze substitution was performed in a Leica AFS2 using 2% osmium tetroxide, 0.1% uranyl acetate and 5% water in acetone. The temperature was raised from –90 to 20 °C at a rate of 5 °C h^–1^. After three washes with acetone, samples were infiltrated and embedded in Epon and polymerized as described above. Ultrathin sections were observed under a Tecnai T12 Electron Microscope.

### Bulk RNA-seq

To address cellular responses to the different drug treatments by RNA-seq, we used two *APOB*^*−/−*^ lines from two donors. Organoids from one well of a 12-well plate were harvested and washed in cold AdvDMEM+++. Organoid pellets were lysed in 1 ml of TRIzol Reagent (Thermo Fisher) and subsequently snap-frozen in liquid nitrogen. RNA was extracted according to the manufacturer’s protocol. RNA integrity was measured using the Agilent RNA 6000 Nano kit with the Agilent 2100 Bioanalyzer, and RNA concentrations were determined using the Qubit RNA HS Assay Kit (Thermo Fisher). RNA integrity number values of RNA samples were typically 9.5–10.0 and never <9.0. RNA libraries were prepared with the TruSeq Stranded messenger RNA polyA kit and paired-end (2 × 50 base pairs) sequenced on an Illumina NextSeq 2000. Reads were mapped to the human GRCh37 genome assembly. Differential gene expression analysis was performed using the DESeq2 package in RStudio (v.2022.02.2). Considered log_2_ fold changes (FC) and significance (*P* values, Wald test) are indicated throughout the paper. Data visualization was performed using the packages ggplot2, ComplexHeatmap and EnhancedVolcano in RStudio, or manually plotted using GraphPad Prism (v.8.2.0). The coefficient of determination (*R*^2^) was determined either using the ggplot2 package in RStudio or in GraphPad Prism.

### DNL assay

To address the contribution of DNL-generated lipids to the steatosis phenotypes of *APOB*^*−/−*^ organoids, we used isotope profiling with [U-^13^C]-glucose (Sigma-Aldrich)—that is, glucose in which all six ^12^C atoms are replaced by ^13^C. To this end, we used a base medium consisting of SILAC Advanced DMEM/F-12 Flex medium (no glucose, no phenol red, Thermo Fisher) supplemented with [U-^13^C]-glucose (17.5 mM, which is identical to the glucose concentration present in HEP medium) and with the addition of 700 μM l-arginine (Sigma-Aldrich), 500 μM l-lysine (Sigma-Aldrich), 1× GlutaMAX, 10 mM HEPES and 100 U ml^–1^ penicillin/streptomycin solution. To this medium we added all components present in HEP medium, with the exception of 15% RSPO1-CM (which we found not essential for hepatocyte growth, nor did its absence influence steatosis phenotypes; Supplementary Fig. 4c), to avoid the presence of unlabeled glucose from this medium in the culture medium. Organoids (both WT and *APOB*^*−/−*^) were plated into fresh BME droplets and cultured in this medium for 1, 3 or 5 days. Organoids were harvested in cold SILAC medium, washed twice with the same medium and dry organoid pellets were snap-frozen in liquid nitrogen. Lipids were solubilized in water/methanol/chloroform (0.8/2.0/1.0, v/v/v) and, after removal of the protein precipitate, the extract was dried under nitrogen. Total cellular lipids were subsequently hydrolyzed in 0.3 M NaOH in 90% MeOH at 75 °C for 1 h. Unsaponifiable lipids were removed by washing twice with two volumes of hexane, followed by acidification of the hydrolysate. FFAs were then extracted by washing twice more with two volumes of hexane. The latter two hexane fractions were combined, dried under nitrogen and analyzed by liquid chromatography–mass spectrometry (see below). Fatty acids whose mass had shifted by a minimum of +4 Da from the monoisotopic (all ^12^C) mass were considered as having been synthesized de novo. Shifts of 2 and 3 Da were excluded, as this typically reflects chain elongation from pre-existing fatty acids.

### Lipidomics

Organoids (all samples plated at the same density) were cultured for 3 days without changing the medium. Then, the medium (supernatant) from one well of a 12-well plate was collected, spun down to remove cell debris and snap-frozen in liquid nitrogen. Organoids from the same well were harvested in cold AdvDMEM+++, washed twice with cold AdvDMEM+++ and dry organoid pellets were snap-frozen in liquid nitrogen. Lipids from cell pellets and medium, as well as blank medium, were extracted with chloroform and methanol using stable-isotope-labeled SPLASH II LIPIDOMIX Mass Spec Standard (Avanti Polar Lipids) for quantification as required^67^. Lipid extracts were kept frozen under a nitrogen atmosphere until analysis. Neutral lipid analysis was performed on an ACQUITY Premier BEH C18 column (130 Å, 1.7 µm, 2.1 × 100 mm^2^, Waters Corporation). Elution was performed at 60 °C using a binary gradient from (A) methanol:water (50:50, v/v) to (B) methanol:isopropanol:ethyl acetate (80:12:8, v/v/v). Both solvents contained 25 mM ammonium formate. Gradient composition was (time, %B): (0, 60); (2.5, 100); (8, 100); (8.1, 60); (10, 60) and the flow rate was kept constant at 0.4 ml min^–1^. The column effluent was introduced into a X500R QToF-type mass spectrometer (Sciex), either via an atmospheric pressure chemical ionization source (APCI) or a heated electrospray ionization source (HESI), both operated in the positive-ion mode. Data from the APCI interfaced runs were used to determine oxysterols, sterols, sterol esters and total TAG content. Data from the subsequent HESI run were used to determine molecular species composition of TAG. Data analysis was performed using the XCMS package in R (v.4.2.0). The fatty acid composition of TAG was elucidated with MS-DIAL, using the default setting for identification confidence (80%)^68^. PCA was performed using the nonlinear iterative partial least-squares (nipals) method with pareto scaling. FFAs were analyzed in negative HESI mode, in the range 150–500 amu with 0.1 s accumulation time. FFAs were eluted from the BEH C18 column mentioned above, using a 7-min gradient from 87.5 to 100% ACN/MeOH (6:4, v/v) in water containing 25 mM ammonium formate.

### Statistical analysis

No statistical methods were used to predetermine sample size. The experiments were not randomized, and the investigators were not blinded to sample allocation during experiments and outcome assessment. Quantitative data are presented either as box-and-whisker plots—with the box spanning the interquartile range, the center line indicating the median value and the whiskers ranging from minimum to maximum values—or as bar plots where mean ± s.d. is depicted. Statistical analyses were performed in GraphPad Prism using two-tailed *t*-tests (with a nested design where appropriate), with the exception of data derived from bulk RNA-seq for which the Wald test used in DESeq2 analysis was applied. The coefficient of determination (*R*^2^), determined either with GraphPad Prism or RStudio (ggplot2 package), was calculated to determine correlation between the different steatosis models regarding drug responses (lipid scores) and correlation between transcriptomic drug responses. Sample sizes (*n*), statistical tests, *P* values and considered statistical significance are indicated in the figure legends.

### Reporting summary

Further information on research design is available in the Nature Portfolio Reporting Summary linked to this article.

## Online content

Any methods, additional references, Nature Portfolio reporting summaries, source data, extended data, supplementary information, acknowledgements, peer review information; details of author contributions and competing interests; and statements of data and code availability are available at 10.1038/s41587-023-01680-4.

### Supplementary information

Supplementary Information Supplementary Figs. 1–15 and Tables 1–3.

Reporting Summary

Supplementary Video 1 Live imaging of *APOB*^*−/−*^ organoids treated with ACC_i for 3 days.

## Data Availability

RNA-seq data are deposited in Gene Expression Omnibus under accession no. GSE221705. The human genome GRCh37 can be accessed at https://www.ncbi.nlm.nih.gov/assembly/GCF_000001405.25.
