# Supplementary material for: Engineered human hepatocyte organoids enable CRISPR-based target discovery and drug screening for steatosis
Source: Nat Biotechnol. 2023 Feb 23;41(11):1567–81. doi: 10.1038/s41587-023-01680-4 (PMC10635827; doi:10.1038/s41587-023-01680-4)
Supplement: Supplementary file 1 — Supplementary Figs. 1–15 and Tables 1–3. [file 41587_2023_1680_MOESM1_ESM.pdf]

nature biotechnology

Article https://doi.org/10.1038/s41587-023-01680-4

Engineered human hepatocyte organoids enable CRISPR-based target discovery and drug screening for steatosis

In the format provided by the authors and unedited

Table of contents

Supplementary Figures 1-15

Supplementary Tables 1-3

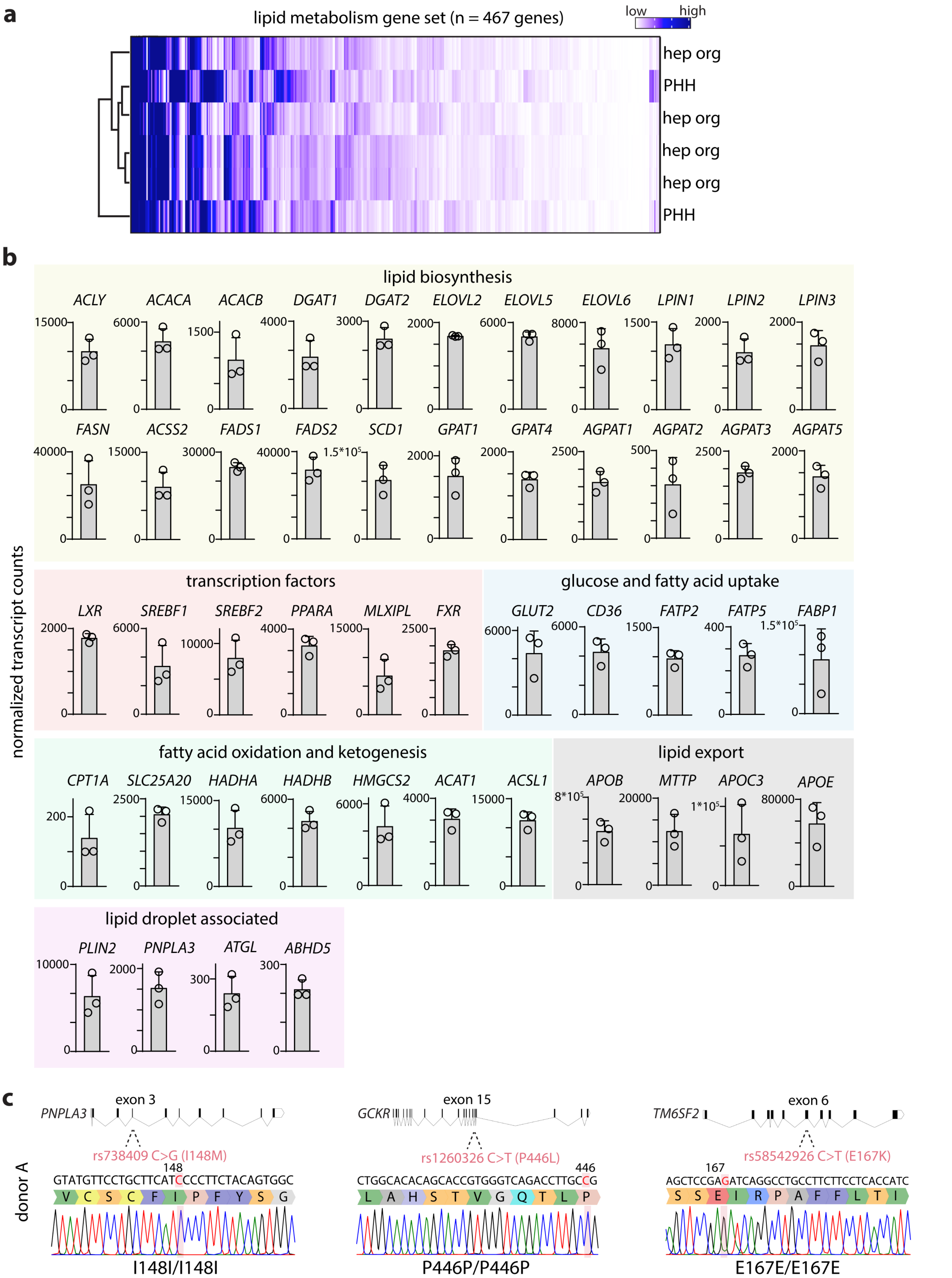

Supplementary Figure 1. Lipid metabolism profiles and NAFLD risk SNP genotyping of human fetal hepatocyte organoids.

a, Heatmap displaying the expression profiles of 467 lipid metabolism genes in human fetal hepatocyte organoids (hep org) and primary human hepatocytes (PHH). Transcript levels within each sample are visualized on a scale from low (0 transcripts, white) to high (6000 transcripts, dark blue).

b, Expression of genes related to different processes encompassing lipid metabolism in human fetal hepatocyte organoids. Normalized transcripts from bulk RNA-sequencing are plotted with the mean + SD. Dots represent expression levels in organoid lines from 3 donors.

c, Sanger trace sequencing examples of an organoid line (donor A) for the top NAFLD risk SNPs in the *PNPLA3*, *GCKR*, and *TM6SF2* genes, showing the donor to be wild type for all SNPs.

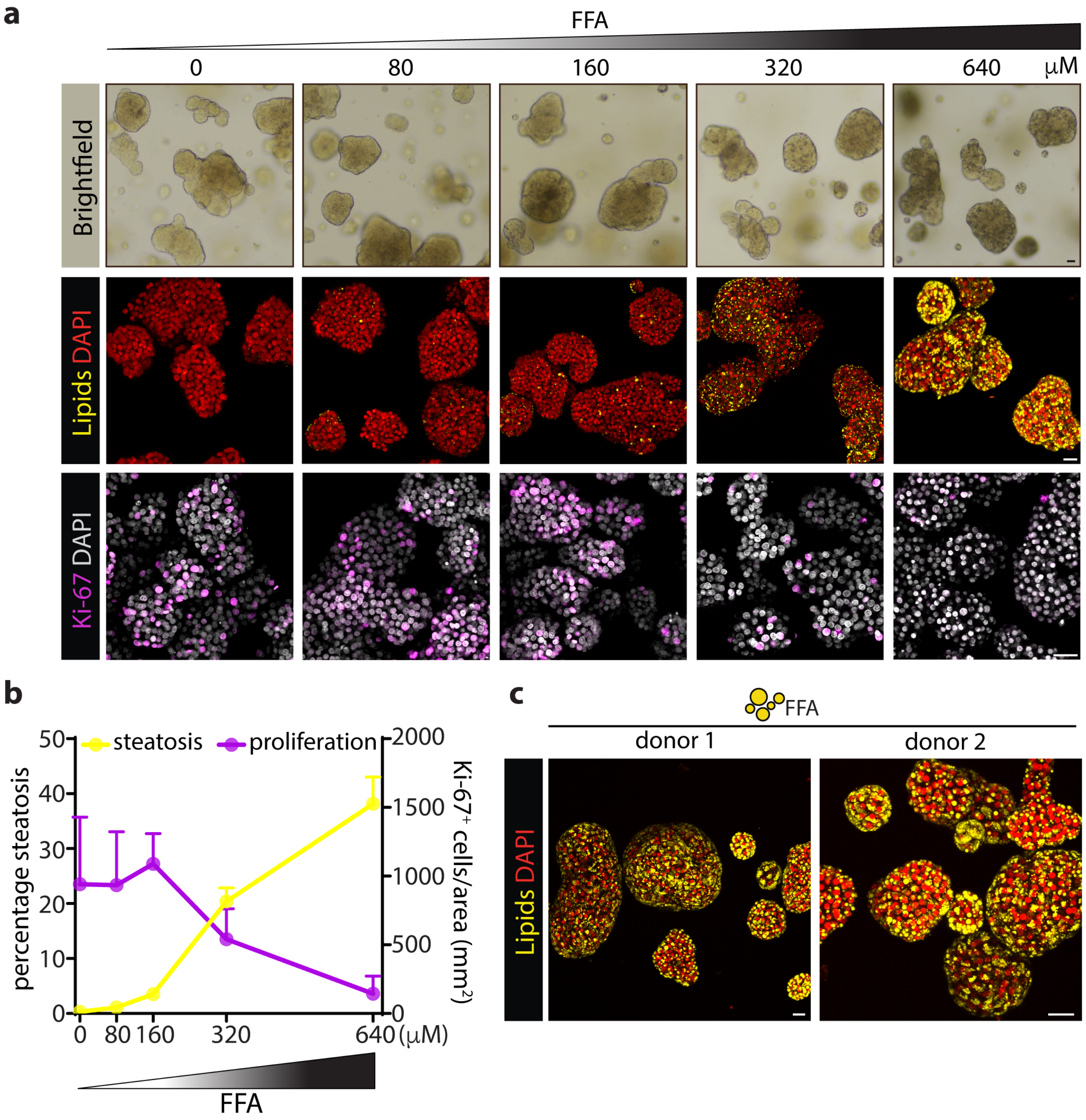

Supplementary Figure 2. Exogenous free fatty acids induce steatosis and impair proliferation.

a, Brightfield images, Nile Red lipid staining, and immunofluorescence staining for Ki-67 of wild type organoids exposed to increasing concentrations of FFAs for 5 days.

b, Quantification of the percentage of steatosis (left y-axis) as well as the amount of Ki-67$^+$ cells/organoid area (right y-axis) in wild type organoids after FFA exposure for 5 days (mean + SD). n = 6 independent replicates from 2 donors (steatosis level), n = 5 independent replicates representative of 2 donors (Ki-67).

c, Nile Red lipid staining of FFA-exposed organoids (500 μM) from 2 donors.

a, **c**, Representative of n = 2 and 6 independent experiments, respectively.

Scale bars, 50 μm (brightfield) and 25 μm (fluorescence) (**a**), 25 μm (**c**).

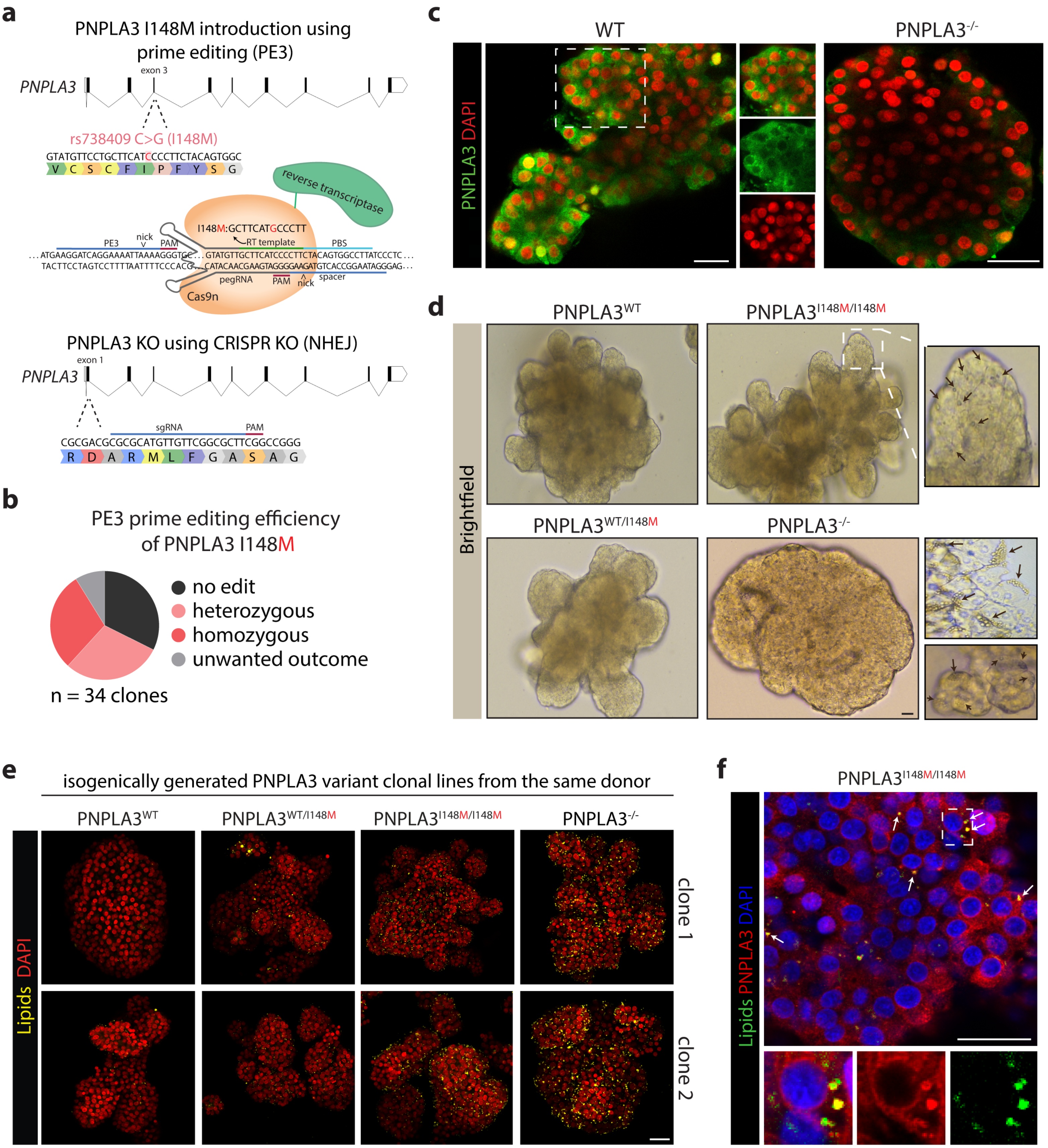

Supplementary Figure 3. Generation of PNPLA3 variant organoids.

a, Strategy to introduce the I148M mutation in the *PNPLA3* gene using PE3 prime editing (top) and the sgRNA used to generate PNPLA3 knock-out organoids (bottom).

b, Assessment of PE3 prime editing efficiency to introduce the I148M mutation.

c, Immunofluorescence staining for PNPLA3 in wild type and *PNPLA3*$^{-/-}$ organoids, confirming the loss of PNPLA3 protein upon knock-out of *PNPLA3*.

d, Brightfield images of PNPLA3 variant organoids. Arrows point at visible clusters of lipid droplets.

e, Nile Red lipid staining of 2 different generated clonal lines per *PNPLA3* genotype, all engineered from the same donor, demonstrating reproducible steatosis levels related to the different *PNPLA3* genotypes.

f, Immunofluorescence staining for PNPLA3 and lipid droplet visualization with BioTracker 488 Green Lipid Droplet Dye in *PNPLA3*$^{I148M/I148M}$ organoids. Arrows highlight the frequent overlap between strong PNPLA3 fluorescent signal and lipid droplets.

c, **d**, **e**, **f**, Representative of n = 3, 6, 6, and 3 independent experiments, respectively.

Scale bars, 25 μm (**c**, **d**, **f**), 50 μm (**e**).

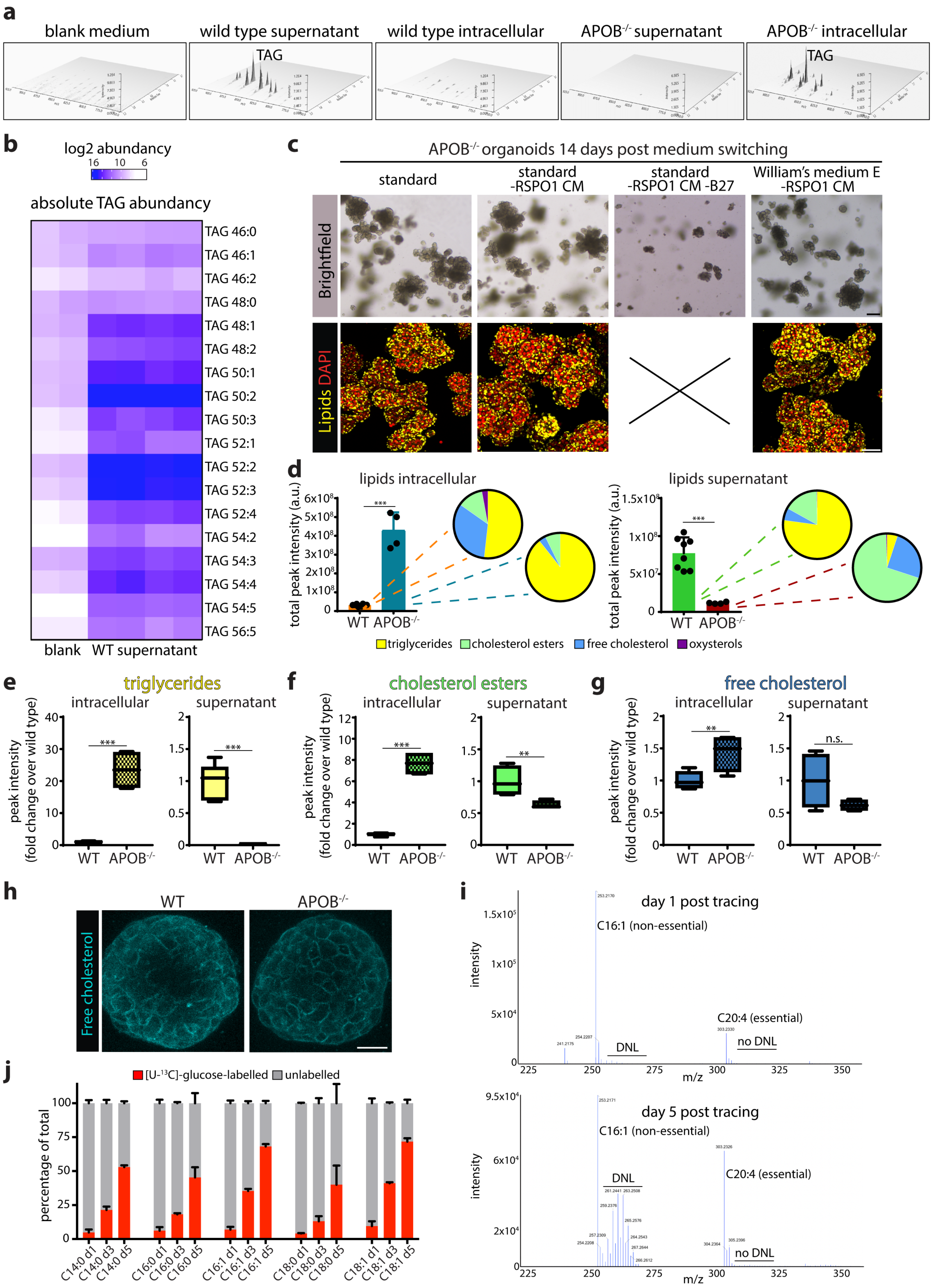

a
blank medium
wild type supernatant
wild type intracellular
APOB-/- supernatant
APOB-/- intracellular
TAG
b
log2 abundancy
16 10 6
absolute TAG abundancy
TAG 46:0
TAG 46:1
TAG 46:2
TAG 48:0
TAG 48:1
TAG 48:2
TAG 50:1
TAG 50:2
TAG 50:3
TAG 52:1
TAG 52:2
TAG 52:3
TAG 52:4
TAG 54:2
TAG 54:3
TAG 54:4
TAG 54:5
TAG 56:5
blank
WT supernatant
c
APOB-/- organoids 14 days post medium switching
standard
standard -RSPO1 CM
standard -RSPO1 CM -B27
William's medium E -RSPO1 CM
Brightfield
Lipids DAPI
d
lipids intracellular
lipids supernatant
total peak intensity (a.u.)
WT APOB-/-
triglycerides
cholesterol esters
free cholesterol
oxysterols
e
triglycerides
f
cholesterol esters
g
free cholesterol
intracellular
supernatant
peak intensity (fold change over wild type)
WT APOB-/-
n.s.
h
WT
APOB-/-
Free cholesterol
i
day 1 post tracing
C16:1 (non-essential)
C20:4 (essential)
DNL
no DNL
day 5 post tracing
intensity
m/z
j
[U-13C]-glucose-labelled
unlabelled
percentage of total
C14:0 d1 C14:0 d3 C14:0 d5
C16:0 d1 C16:0 d3 C16:0 d5
C16:1 d1 C16:1 d3 C16:1 d5
C18:0 d1 C18:0 d3 C18:0 d5
C18:1 d1 C18:1 d3 C18:1 d5

Supplementary Figure 4. Lipidomic characterization of wild type and *APOB*$^{-/-}$ organoids.

a, Surface plots of TAG species detected intracellularly and in the supernatant of wild type organoids and *APOB*$^{-/-}$ organoids, and comparison with blank medium, demonstrating active VLDL secretion in wild type organoids, while this is defective in *APOB*$^{-/-}$ organoids as showcased by the absence of TAG in the medium.

b, Heatmap displaying the absolute abundancy (log2-transformed) of TAG species in the supernatant of 3-day-cultured wild type organoids and comparison with blank medium. Each column is an independent replicate. n = 4 organoid cultures from 2 donors, n = 2 for blank medium.

c, Brightfield images and Nile Red lipid staining of medium withdrawal experiments confirm that withdrawal of putative lipid sources in the medium (*e.g.* RSPO1-conditioned medium (CM)) does not alter the steatosis phenotype of *APOB*$^{-/-}$ organoids. Note that organoids did not survive in the standard -RSPO1 CM -B27 condition and therefore Nile Red lipid staining could not be performed.

d, Quantification of the total neutral lipids (TAG, cholesterol esters, free cholesterol, and oxysterols) detected in wild type and *APOB*$^{-/-}$ organoids intracellularly (left) and in the supernatant (right) (mean + SD). The pie charts indicate the average composition of the different lipid species (not scaled to abundance). n = 4 independent measurements in *APOB*$^{-/-}$ organoid cultures from 2 donors, n = 8 independent measurements in wild type organoid cultures from the same 2 donors. Two-tailed *t*-test: $P < 0.0001$ (***). a.u. = arbitrary units.

e-g, Quantification of the accumulation/reduction of specific lipid species (**e**, triglycerides; **f**, cholesterol esters; **g**, free cholesterol) in *APOB*$^{-/-}$ organoids intracellularly and in the supernatant relative to the amount detected in wild type organoids. Sample sizes as in **d**. Two-tailed *t*-test: triglycerides intracellular *APOB*$^{-/-}$ *vs* WT, $P < 0.0001$ (***); triglycerides supernatant *APOB*$^{-/-}$ *vs* WT, $P < 0.0001$ (***); cholesterol esters intracellular *APOB*$^{-/-}$ *vs* WT, $P < 0.0001$ (***); cholesterol esters supernatant *APOB*$^{-/-}$ *vs* WT, $P = 0.0075$ (**); free cholesterol intracellular *APOB*$^{-/-}$ *vs* WT, $P = 0.0036$ (**); n.s. = not significant.

h, Filipin III staining marking free cholesterol in wild type and *APOB*$^{-/-}$ organoids, demonstrating its predominant presence on the membrane.

i, Representative mass spectra highlighting the selectivity in [U-^{13}C]-glucose incorporation between the non-essential (C16:1) and essential (C20:4) fatty acid at day 1 (top) and day 5 (bottom) post tracing in of *APOB*$^{-/-}$ organoids. Note at day 5 the extensive labelling of DNL-derived C16:1 species, which have a higher m/z due to the ^{13}C (instead of ^{12}C) labelling. Instead, no labelling for C20:4 is seen during the entire tracing period, as this fatty acid is essential and cannot be formed by DNL.

j, Quantification of the percentage of glucose-driven DNL contribution for the five non-essential fatty acids that can be formed by DNL after 1, 3, and 5 days of tracing in *APOB*$^{-/-}$ organoids (mean + SD). n = 2 independent quantifications in *APOB*$^{-/-}$ organoid cultures from 2 donors.

e-g, The box indicates the 25-75th percentiles, the centre line indicates the median and the whiskers indicate minimum and maximum values.

c, **h**, Representative of n = 3 and 2 independent experiments, respectively.

Scale bars, 200 μm (brightfield) and 50 μm (fluorescence) (**c**), 50 μm (**h**).

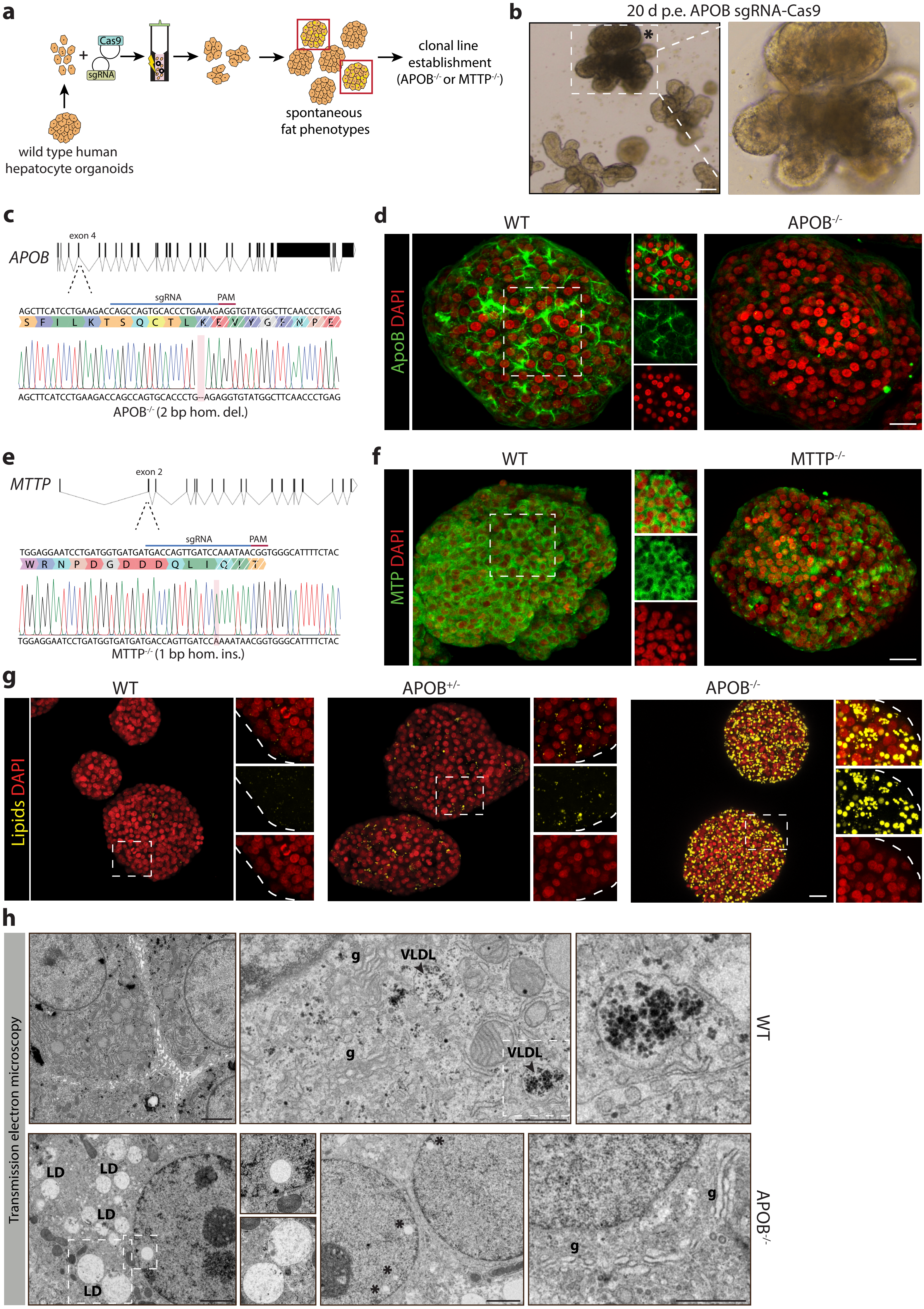
a
Cas9
sgRNA
wild type human hepatocyte organoids
spontaneous fat phenotypes
clonal line establishment (APOB-/- or MTTP-/-)
b
20 d p.e. APOB sgRNA-Cas9
c
APOB
exon 4
sgRNA
PAM
AGCTTCATCCTGAAGACCAGCCAGTGCACCCTGAAAGAGGTGTATGGCTTCAACCCTGAG
S F I L K T S Q C T L K E V Y G F N P E
AGCTTCATCCTGAAGACCAGCCAGTGCACCCTG--AGAGGTGTATGGCTTCAACCCTGAG
APOB-/- (2 bp hom. del.)
d
WT
APOB-/-
ApoB DAPI
e
MTTP
exon 2
sgRNA
PAM
TGGAGGAATCCTGATGGTGATGATGACCAGTTGATCCAAATAACGGTGGGCATTTTCTAC
W R N P D G D D D Q L I Q I T
TGGAGGAATCCTGATGGTGATGATGACCAGTTGATCCAAAATAACGGTGGGCATTTTCTAC
MTTP-/- (1 bp hom. ins.)
f
WT
MTTP-/-
MTP DAPI
g
WT
APOB+/-
APOB-/-
Lipids DAPI
h
Transmission electron microscopy
g
VLDL
g
VLDL
WT
LD
LD
LD
LD
g
g
APOB-/-

Supplementary Figure 5. Characterization of *APOB*- and *MTTP*-mutant human hepatocyte organoids.
a, Strategy to CRISPR engineer human hepatocyte organoids and generate *APOB*$^{-/-}$ or *MTTP*$^{-/-}$ organoid lines.
b, Representative example of an outgrowing organoid culture upon electroporation with APOB-sgRNA/Cas9, demonstrating the spontaneous outgrowth of darker -lipid containing- organoids (highlighted by the asterisk).
c, Schematic of the sgRNA used to generate *APOB* knock-out organoids (top) and a Sanger trace sequencing example of a generated clonal *APOB*$^{-/-}$ line (bottom).
d, Immunofluorescence staining for ApoB in wild type and *APOB*$^{-/-}$ organoids, confirming the loss of ApoB protein upon knock-out of *APOB*.
e, Schematic of the sgRNA used to generate *MTTP* knock-out organoids (top) and a Sanger trace sequencing example of a generated clonal *MTTP*$^{-/-}$ line (bottom).
f, Immunofluorescence staining for MTP in wild type and *MTTP*$^{-/-}$ organoids, confirming the loss of MTP protein upon knock-out of *MTTP*. Note that the signal detected in *MTTP*$^{-/-}$ organoids represents background staining, lacking cytoplasmic specificity.
g, Nile Red lipid staining of human hepatocyte organoids with different *APOB* genotypes (wild type, heterozygous knock-out, or homozygous knock-out).
h, Transmission electron microscopy images of wild type and *APOB*$^{-/-}$ organoids, demonstrating accumulation of lipid droplets (LD) of various sizes in *APOB*$^{-/-}$ organoids. Wild type organoids possess intact VLDL packaging as shown by the VLDL vesicles (VLDL), marked by arrowheads, around the Golgi apparatus (g), while these are absent in *APOB*$^{-/-}$ organoids. Asterisks indicate the presence of lipid droplets in the nucleus of *APOB*$^{-/-}$ organoids.
d, **f**, **g**, **h**, Representative of n = 2, 2, 3, and 2 independent experiments, respectively.
Scale bars, 100 μm (**b**), 25 μm (**d**, **f**), 50 μm (**g**), 500 nm (left) and 100 nm (right) for wild type and all 100 nm for *APOB*$^{-/-}$ **h**).

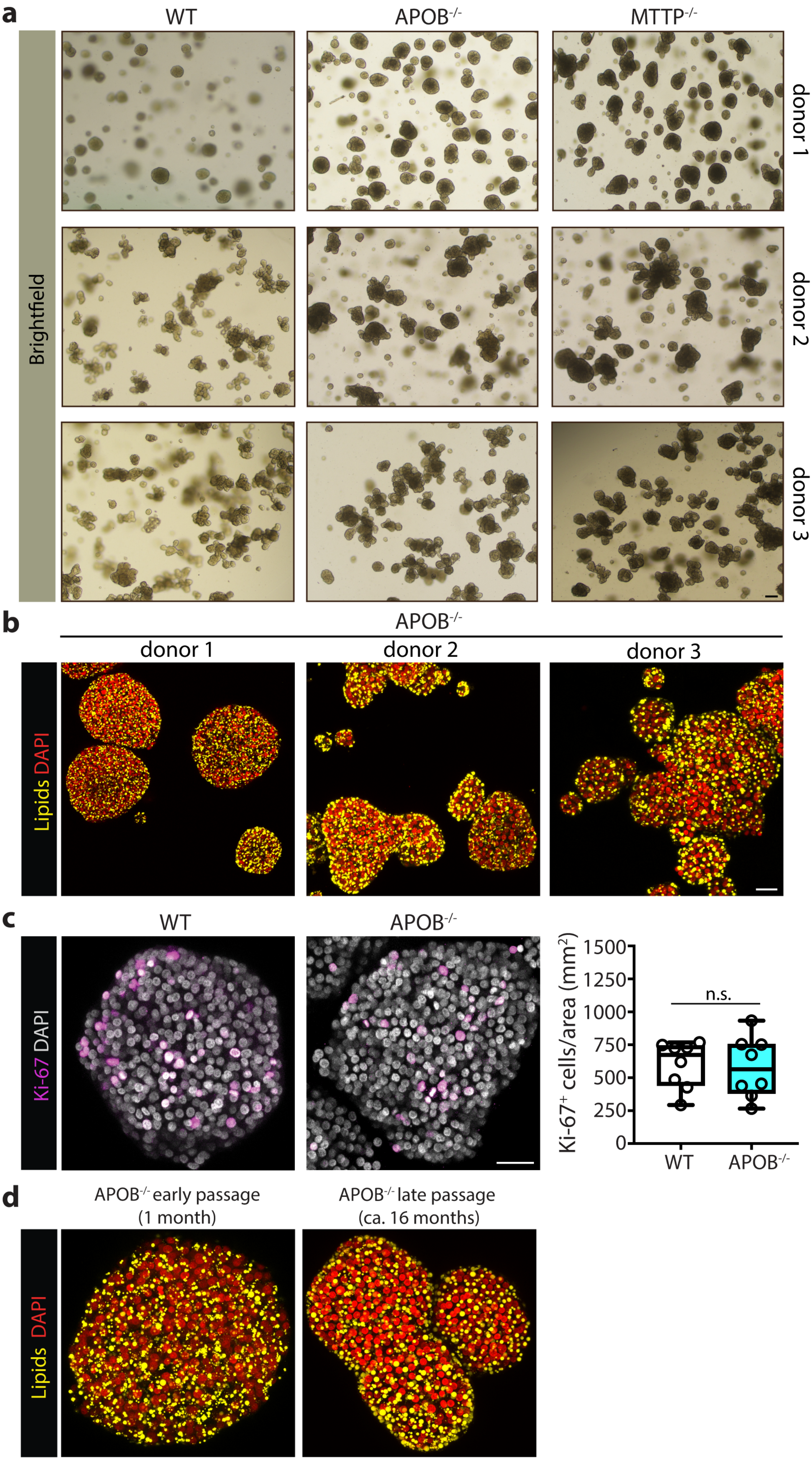

Supplementary Figure 6. *APOB*- and *MTTP*-mutant organoids from multiple donors are proliferative.

a, Brightfield images of WT, *APOB*$^{-/-}$, and *MTTP*$^{-/-}$ organoid cultures from 3 different donors.

b, Nile Red lipid staining of *APOB*$^{-/-}$ organoids from 3 donors, demonstrating reproducible steatosis levels across donors.

c, Immunofluorescence staining for Ki-67 in wild type organoids and *APOB*$^{-/-}$ organoids generated from the same donor. Quantification of the Ki-67^{+} cells/organoid area is shown to the right. n = 8 independent replicates for both conditions representative of 3 donors. Two-tailed *t*-test: n.s. = not significant.

d, Nile Red lipid staining of an early and late passage *APOB*$^{-/-}$ organoid line, demonstrating constant steatosis levels across culture time.

c, The box indicates the 25-75th percentiles, the centre line indicates the median and the whiskers indicate minimum and maximum values.

a, **b**, **c**, **d**, Representative of n = 9, 9, 2, and 3 independent experiments, respectively.

Scale bars, 200 μm (**a**),100 μm (**b**), 25 μm (**c**, **d**).

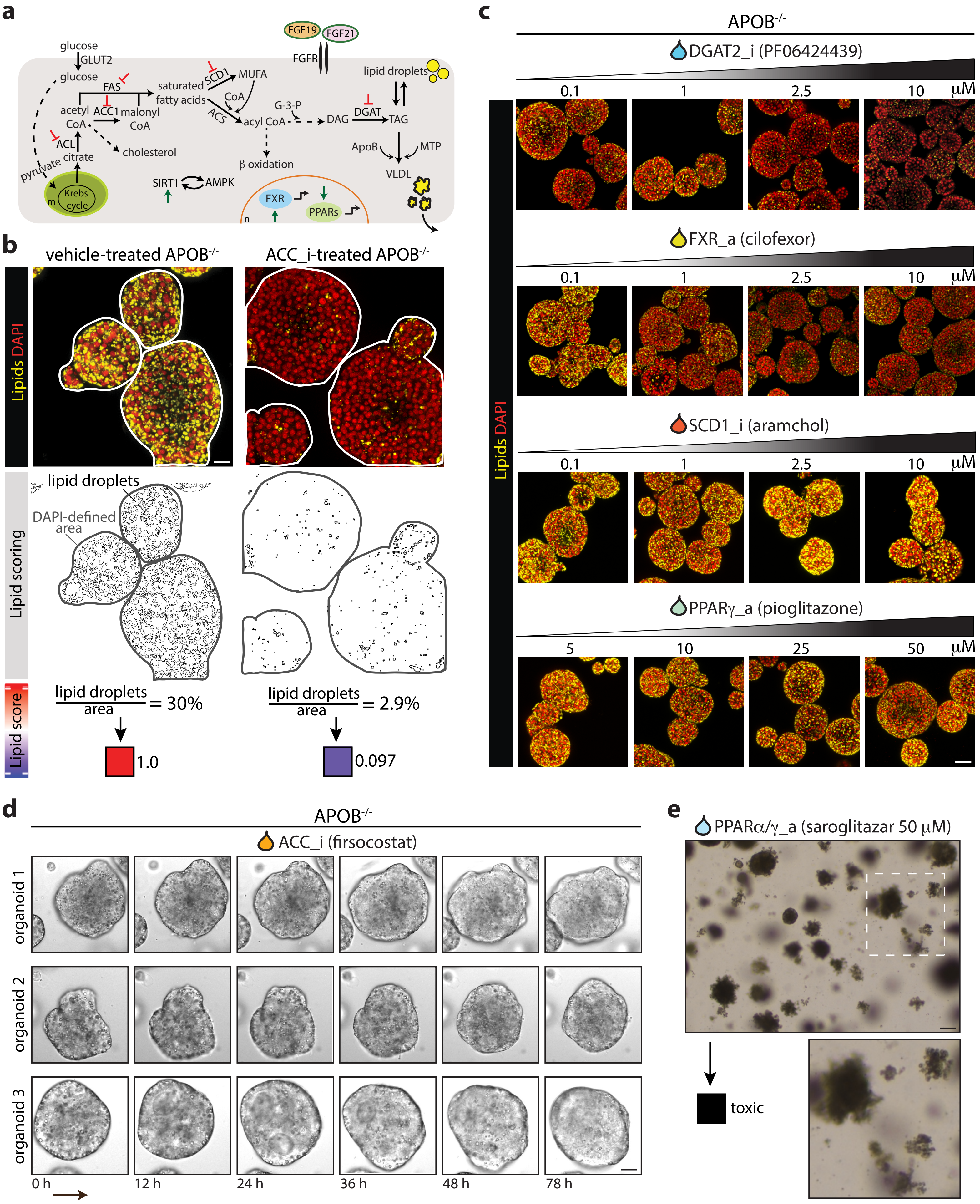

Supplementary Figure 7. NAFLD drug screening using a lipid scoring system.

a, Schematic representation of the hepatocyte targets of some of the evaluated NAFLD drug candidates.

b, Example of the lipid scoring system in *APOB*$^{-/-}$ organoids treated with vehicle or ACC_i. The area of a Z-projected organoid is assessed and outlined based on the presence of DAPI signal (nuclei). The Z-stack is converted to a binary image based on a set threshold from the fluorescent signal from the Nile Red lipid staining. The area occupancy of the particles (lipid droplets) within the organoid is subsequently calculated within the nuclei (DAPI)-defined area. The lipid score represents a relative score ranging from 0 (blue) to 1 (red), where the lipid score of vehicle-treated steatosis organoids per model is set to 1 and those of wild type organoids to 0.

c, Nile Red lipid staining showing the dose responses in *APOB*$^{-/-}$ organoids of the effective drugs DGAT2_i and FXR_a, and ineffective drugs SCD1_i and PPARγ_a.

d, Brightfield images of a live-imaging session of *APOB*$^{-/-}$ organoids upon treatment with ACC_i during 78 hours of treatment. See also **Supplementary Video 1**.

e, Example of a drug exposure classified as toxic. Note the obvious disintegration of the *APOB*$^{-/-}$ organoids upon 7 day-treatment with 50 μM saroglitazar.

b, **c**, **d**, **e**, Representative of n = 4, 4, 3, and 4 independent experiments, respectively.

Scale bars, 50 μm (**b**, **d**),100 μm (**c**), 200 μm (**e**).

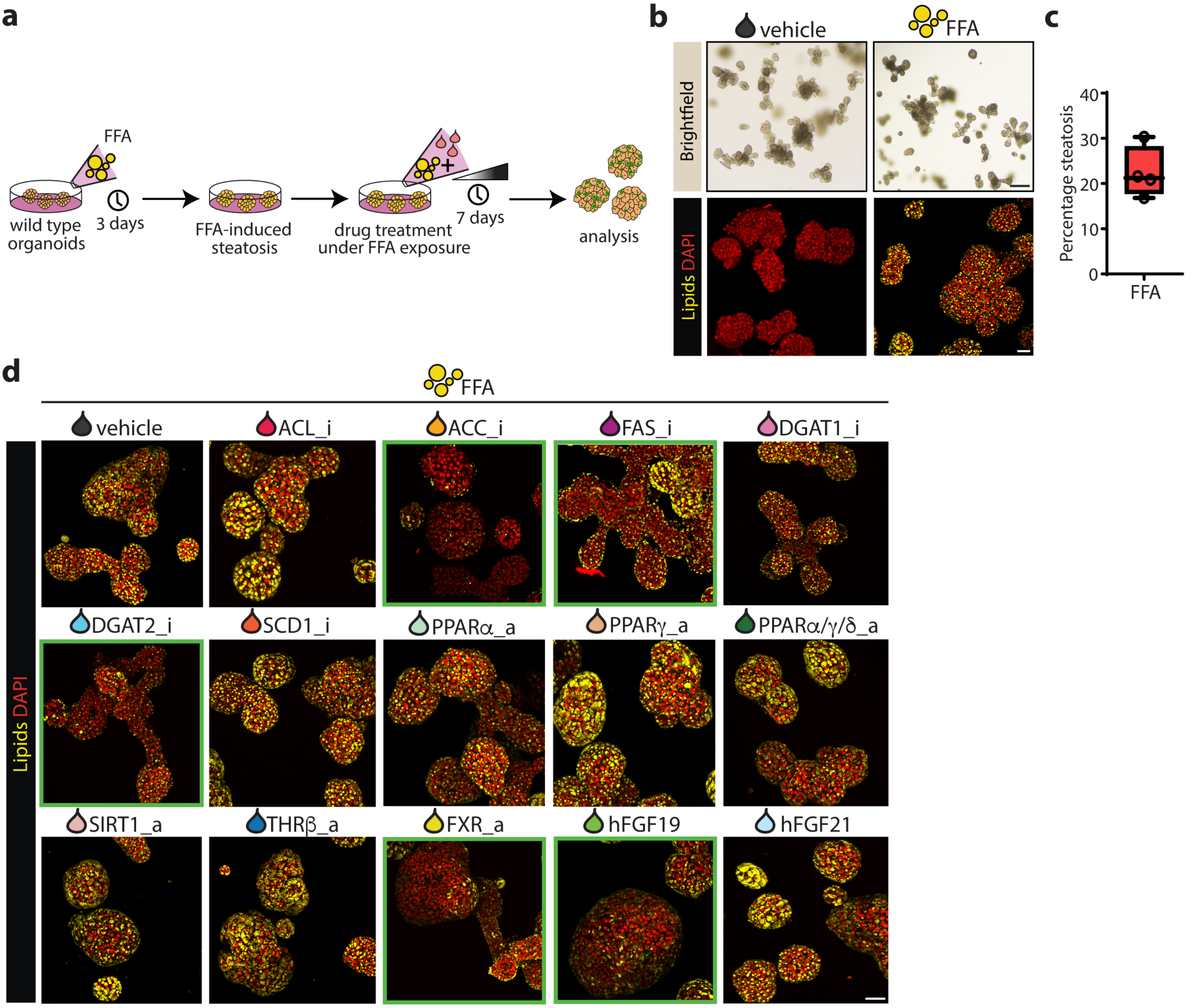

Supplementary Figure 8. Drug responses of FFA-induced steatosis organoids.

a, Strategy to perform drug screening in FFA-induced steatosis organoids.

b, Brightfield images and Nile Red lipid staining of organoids treated with vehicle or FFAs (500 μM) for 3 days.

c, Quantification of the percentage of steatosis after FFA exposure for 3 days prior to drug treatment. n = 4 independent replicates representative of 2 donors.

d, Nile Red lipid staining of the FFA-induced steatosis organoids after treatment with different drugs for 7 days. Green boxes highlight steatosis-reducing effects.

c, The box indicates the 25-75th percentiles, the centre line indicates the median and the whiskers indicate minimum and maximum values.

b, **d**, Representative of n = 2 independent experiments using 2 donors.

Scale bars, 200 μm (brightfield) and 50 μm (fluorescence) (**b**), 50 μm (**d**).

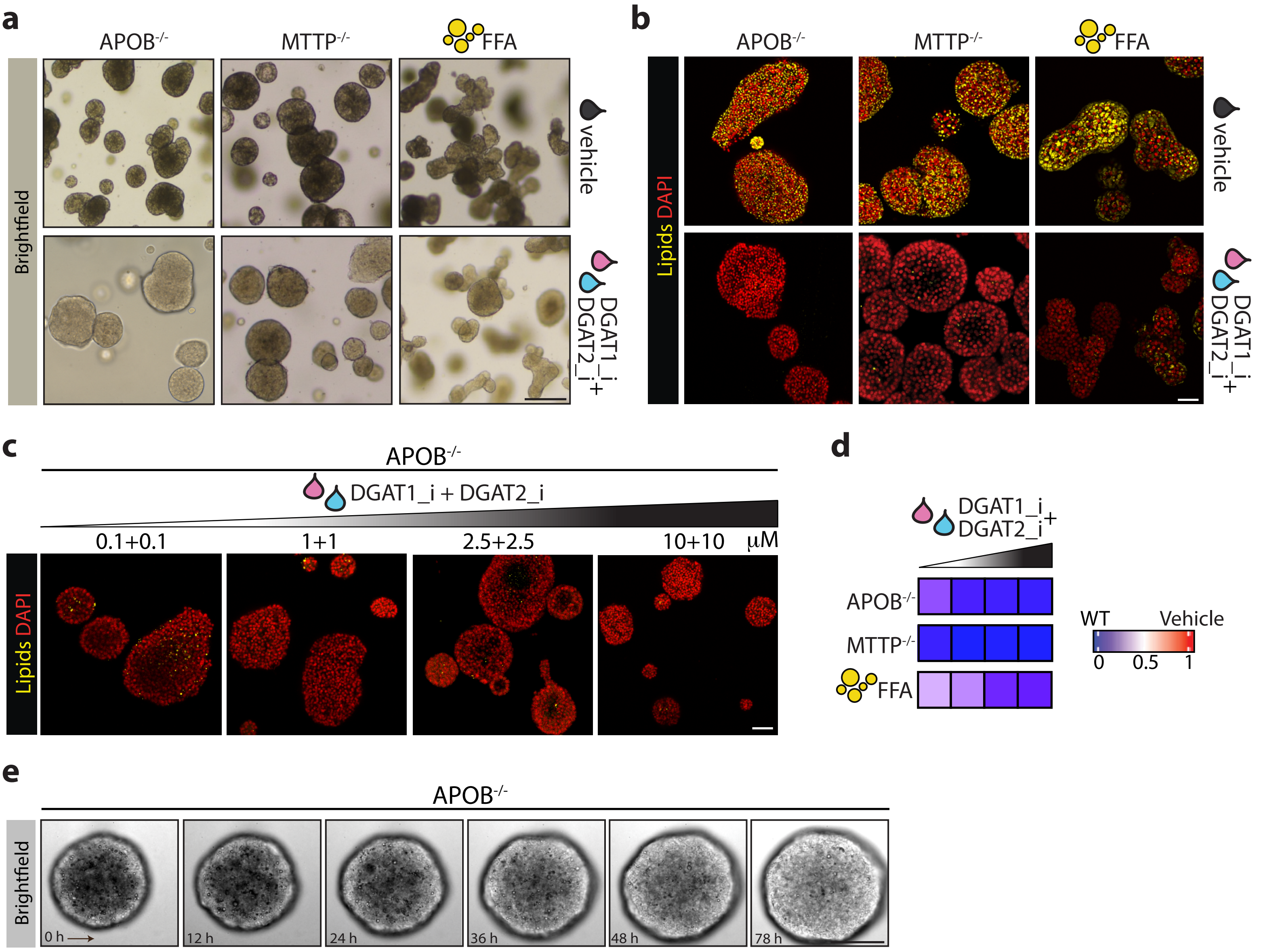

Supplementary Figure 9. Synergistic steatosis-reducing effect of combined DGAT1 and DGAT2 inhibition.

a, Brightfield images of genetic steatosis (*APOB*$^{-/-}$ and *MTTP*$^{-/-}$) and FFA-induced steatosis organoids after treatment with combined DGAT1_i + DGAT2_i (2.5 μM + 2.5 μM) or vehicle.

b, Nile Red lipid staining of genetic steatosis (*APOB*$^{-/-}$ and *MTTP*$^{-/-}$) and FFA-induced steatosis organoids after treatment with combined DGAT1_i + DGAT2_i (2.5 μM + 2.5 μM) or vehicle.

c, Nile Red lipid staining showing the dose responses in *APOB*$^{-/-}$ organoids of combined DGAT1_i + DGAT2_i treatment.

d, Lipid score analyses of the genetic steatosis (*APOB*$^{-/-}$ and *MTTP*$^{-/-}$) and FFA-induced steatosis organoids upon treatment with increasing doses of DGAT1_i + DGAT2_i.

e, Brightfield images of a live-imaging session of *APOB*$^{-/-}$ organoids upon treatment with DGAT1_i + DGAT2_i during 78 hours of treatment.

a, **b**, **c**, **e**, Representative of n = 4, 4, 4, and 3 independent experiments, respectively.

Scale bars, 200 μm (**a**), 50 μm (**b**, **c**),100 μm (**e**).

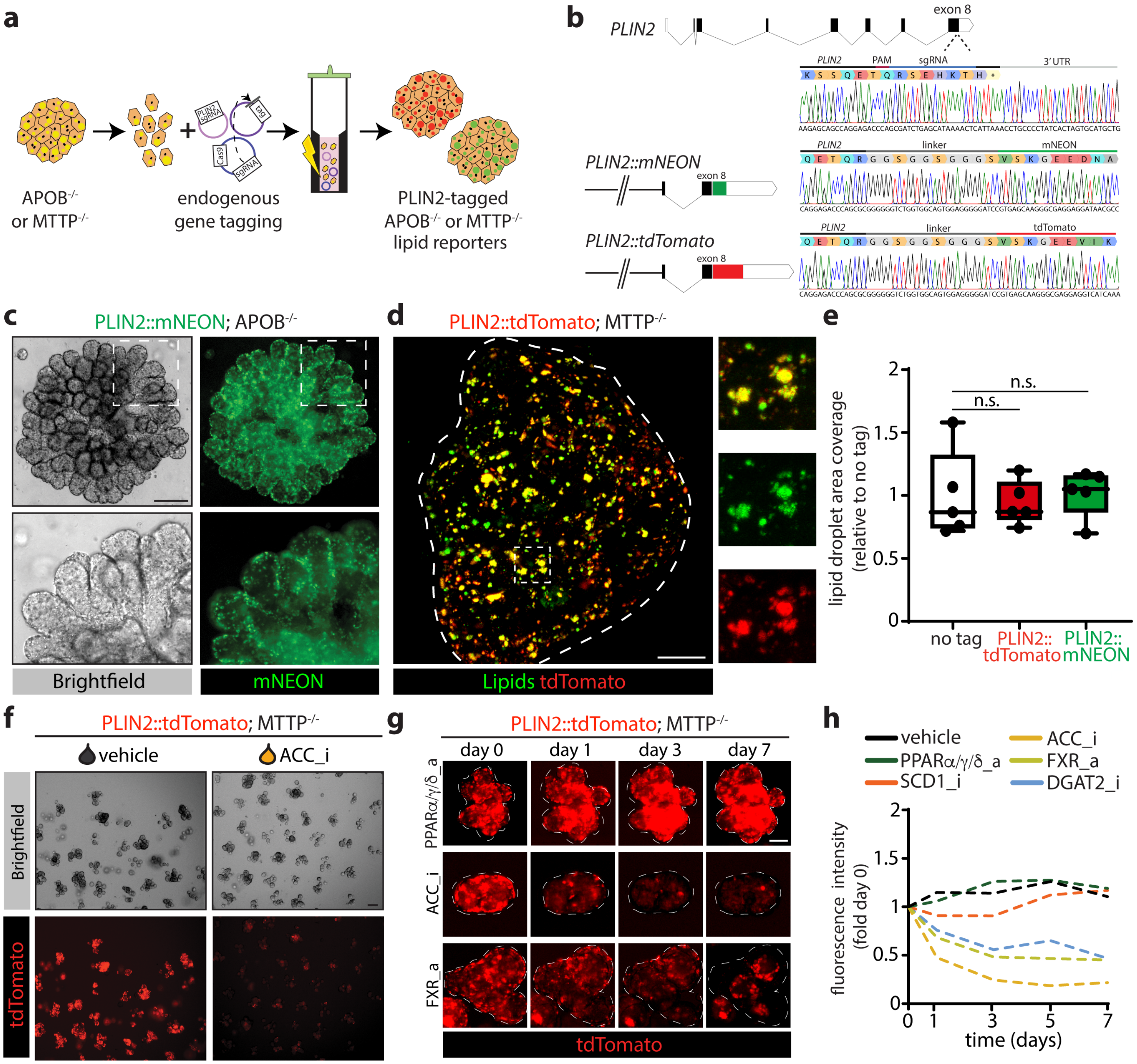

Supplementary Figure 10. Development of PLIN2 lipid reporter organoids.

a, Schematic representation of the workflow to generate endogenous PLIN2-tagged lipid reporter organoids.

b, Sanger trace sequences of *PLIN2::mNEON* and *PLIN2::tdTomato* reporter lines, confirming precise integration of the fluorescent tag at the C-terminus of *PLIN2*.

c, Brightfield and fluorescence images of a *PLIN2::mNEON; APOB*$^{-/-}$ reporter organoid.

d, Fluorescence image showing co-staining of the tdTomato signal from *PLIN2::tdTomato; MTTP*$^{-/-}$ organoids with the lipid droplet signal marked by BioTracker 488 Green Lipid Droplet Dye.

e, Quantification of the lipid droplet area coverages in *PLIN2::tdTomato; MTTP*$^{-/-}$ and *PLIN2::mNEON; MTTP*$^{-/-}$ relative to untagged organoids, originating from the parental *MTTP*$^{-/-}$ line. n = 5 independent replicates per genotype. Two-tailed *t*-test: n.s. = not significant.

f, Brightfield and fluorescence images of a *PLIN2::tdTomato; MTTP*$^{-/-}$ organoid culture treated with ACC_i or vehicle for 5 days.

g, Fluorescence images of *PLIN2::tdTomato; MTTP*$^{-/-}$ organoids treated with different drugs over a 7 day window, demonstrating the reduction/loss of PLIN2 signal upon steatosis-reducing effects of ACC_i and FXR_a.

h, Quantification of drug effects using the fluorescent signal from the *PLIN2::tdTomato; MTTP*$^{-/-}$ reporter as a read-out, using both previously identified steatosis-reducing drugs (ACC_i, DGAT2_i, FXR_a) and drugs with no positive effects (SCD1_i, PPARα/γ/δ_a). The mean response of n = 2 independent measurements is shown.

e, The box indicates the 25-75th percentiles, the centre line indicates the median and the whiskers indicate minimum and maximum values.

c, **d**, **f**, **g**, Representative of n = 3, 3, 2, and 2 independent experments, respectively.

Scale bars, 100 μm (**c**), 50 μm (**d**, **f**), 20 μm (**g**).

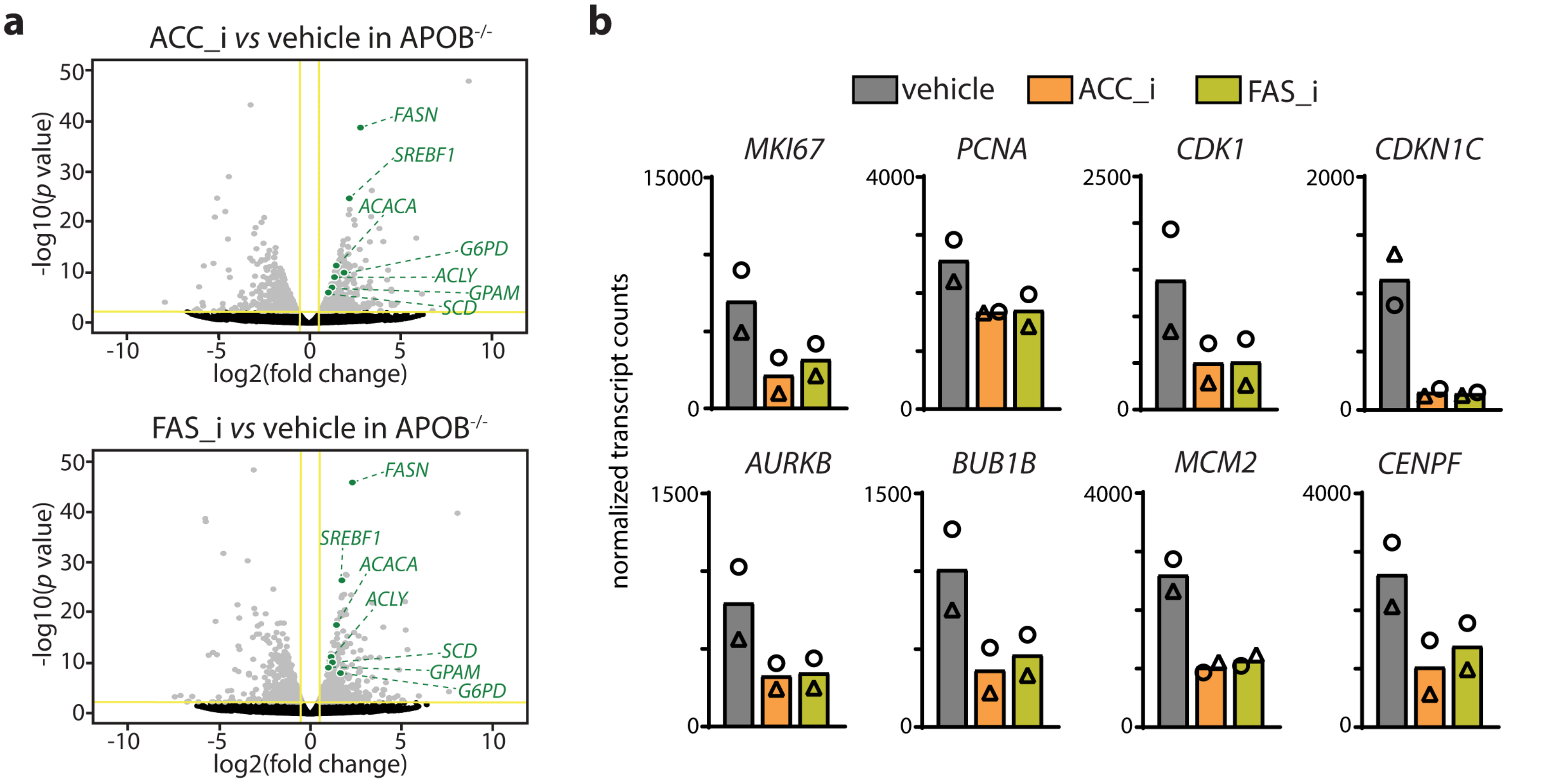

Supplementary Figure 11. Overcompensation by lipogenic enzyme induction and impaired proliferation caused by inhibition of ACC and FAS.

a, Volcano plots showing differential gene expression after treatment with ACC_i (top) and FAS_i (bottom) relative to vehicle-treated *APOB*$^{-/-}$ organoids. Grey dots indicate DEGs (|log2FC| > 0.5, $P < 0.005$ (Wald test)). Annotated genes highlight the counterintuitive induction of many lipogenic genes.

b, Bar plots (mean is shown) demonstrating the reduced expression of several genes implicated in DNA replication and the cell cycle after treatment with ACC_i and FAS_i. n = 2 independent replicates with each symbol representing the expression in an *APOB*$^{-/-}$ line from 2 donors.

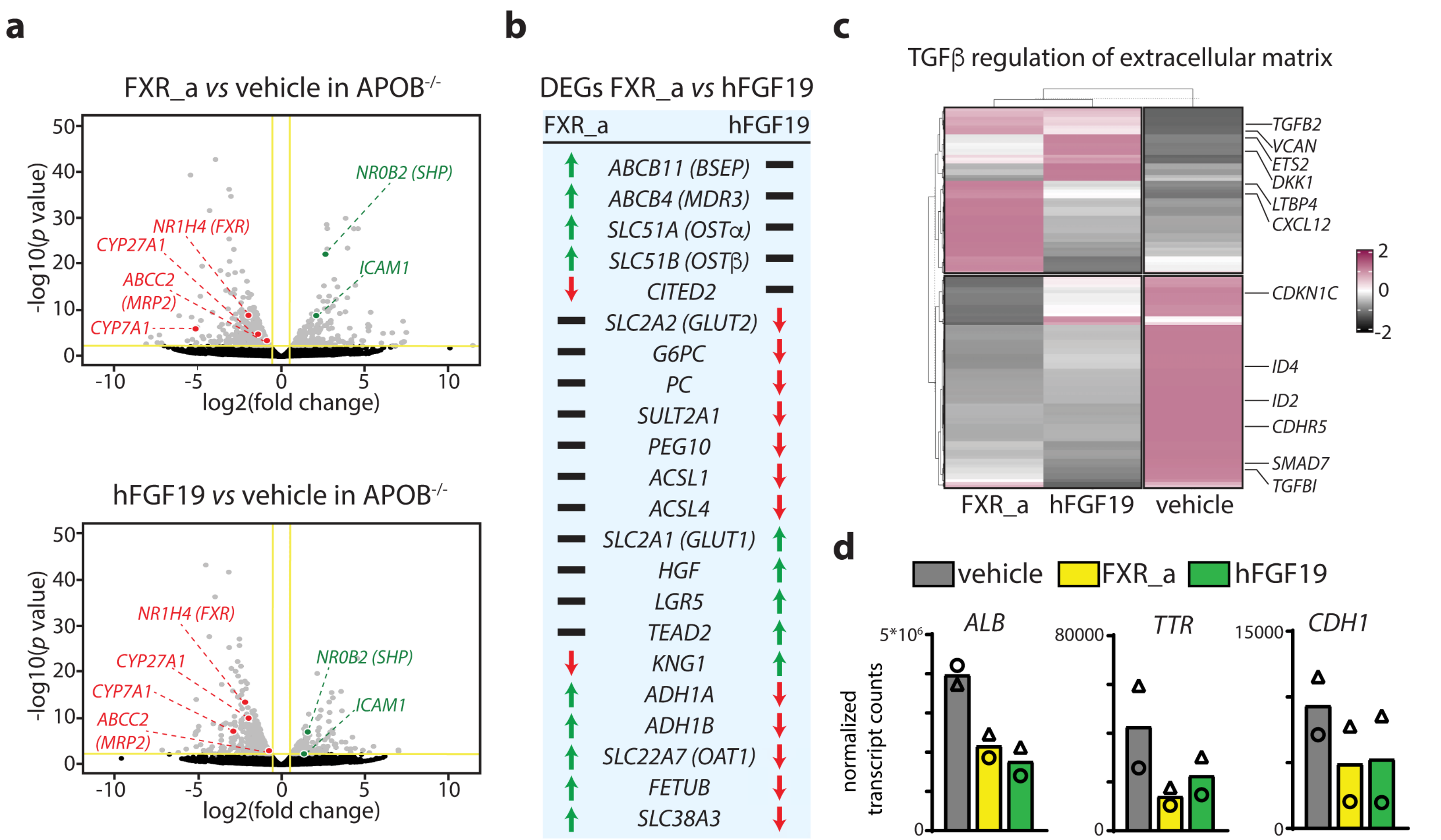

Supplementary Figure 12. FXR activation and hFGF19 treatment regulate bile acid homeostasis and induce EMT-like traits.

a, Volcano plots showing differential gene expression after treatment with FXR_a (top) and hFGF19 (bottom) relative to vehicle-treated *APOB*$^{-/-}$ organoids. Grey dots indicate DEGs (|log2FC| > 0.5, $P < 0.005$ (Wald test)). Annotated genes highlight typical FXR target genes.

b, List of unique DEGs found in either FXR_a- or hFGF19-treated *APOB*$^{-/-}$ organoids. Green arrows denote upregulation, red arrows indicate downregulation, black lines indicate no significant change in expression.

c, Heatmap displaying the expression of genes related to TGFβ regulation of extracellular matrix after treatment with FXR_a and hFGF19 relative to vehicle-treated *APOB*$^{-/-}$ organoids. Row Z-scores are plotted.

d, Bar plots (mean is shown) demonstrating reduced expression of hepatocyte markers (*ALB*, *TTR*) and the epithelial marker *CDH1* after treatment with FXR_a and hFGF19. n = 2 independent replicates with each symbol representing the expression in an *APOB*$^{-/-}$ line from 2 donors.

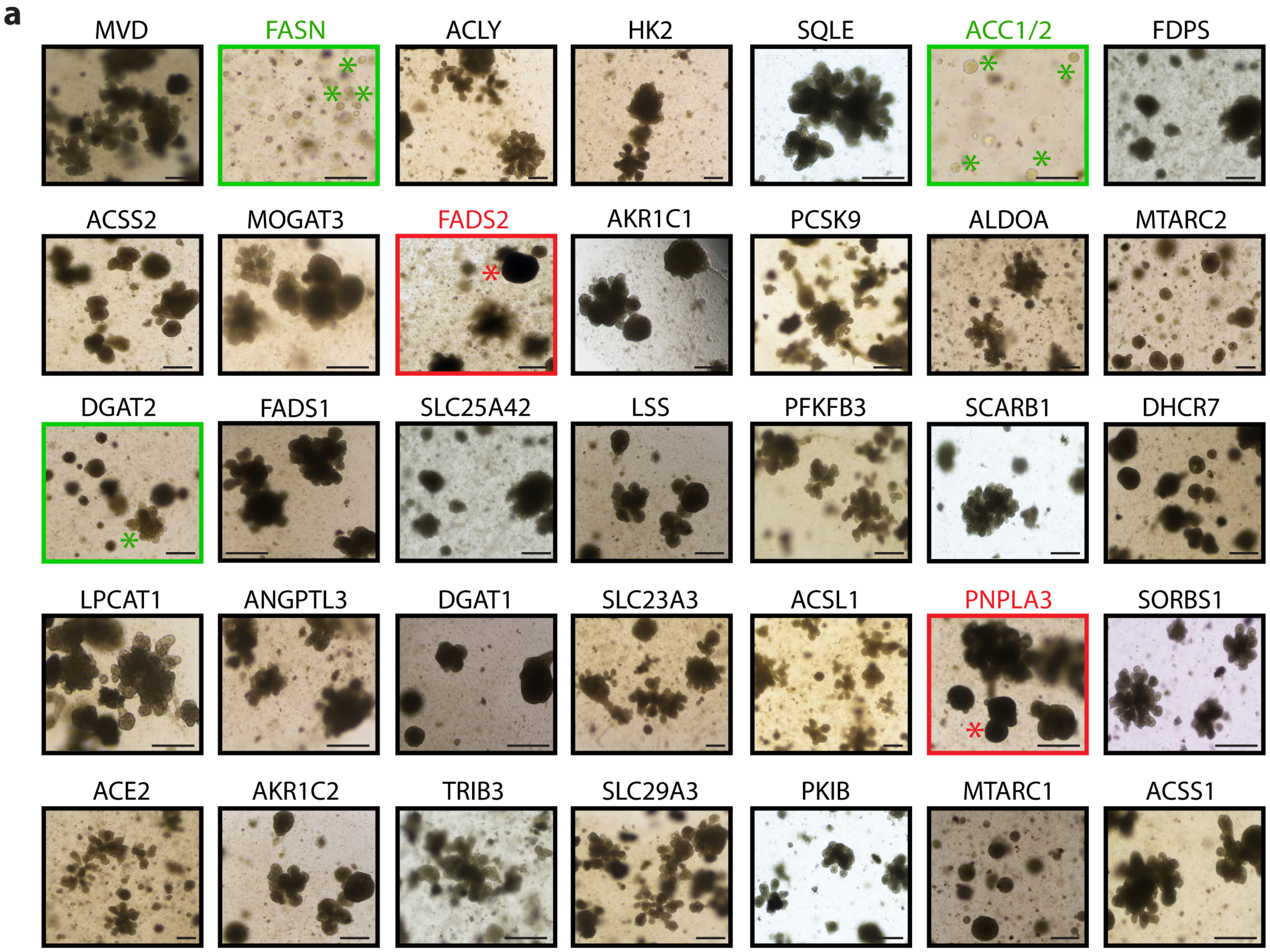

Supplementary Figure 13. CRISPR LOF screening in FatTracer highlights steatosis mediators.
a, Brightfield images of outgrowing FatTracer organoids upon CRISPR LOF of the respective genes. Green boxes highlight appearance of lighter -less lipid containing- organoids, while red boxes highlight appearance of darker -more lipid containing- organoids. Note that differences in organoid morphology are due to the donor-to-donor variability. Representative of n = 2 independent experiments using both *APOB*$^{-/-}$ and *MTTP*$^{-/-}$ organoids from 2 donors as FatTracer. Scale bar, 100 μm.

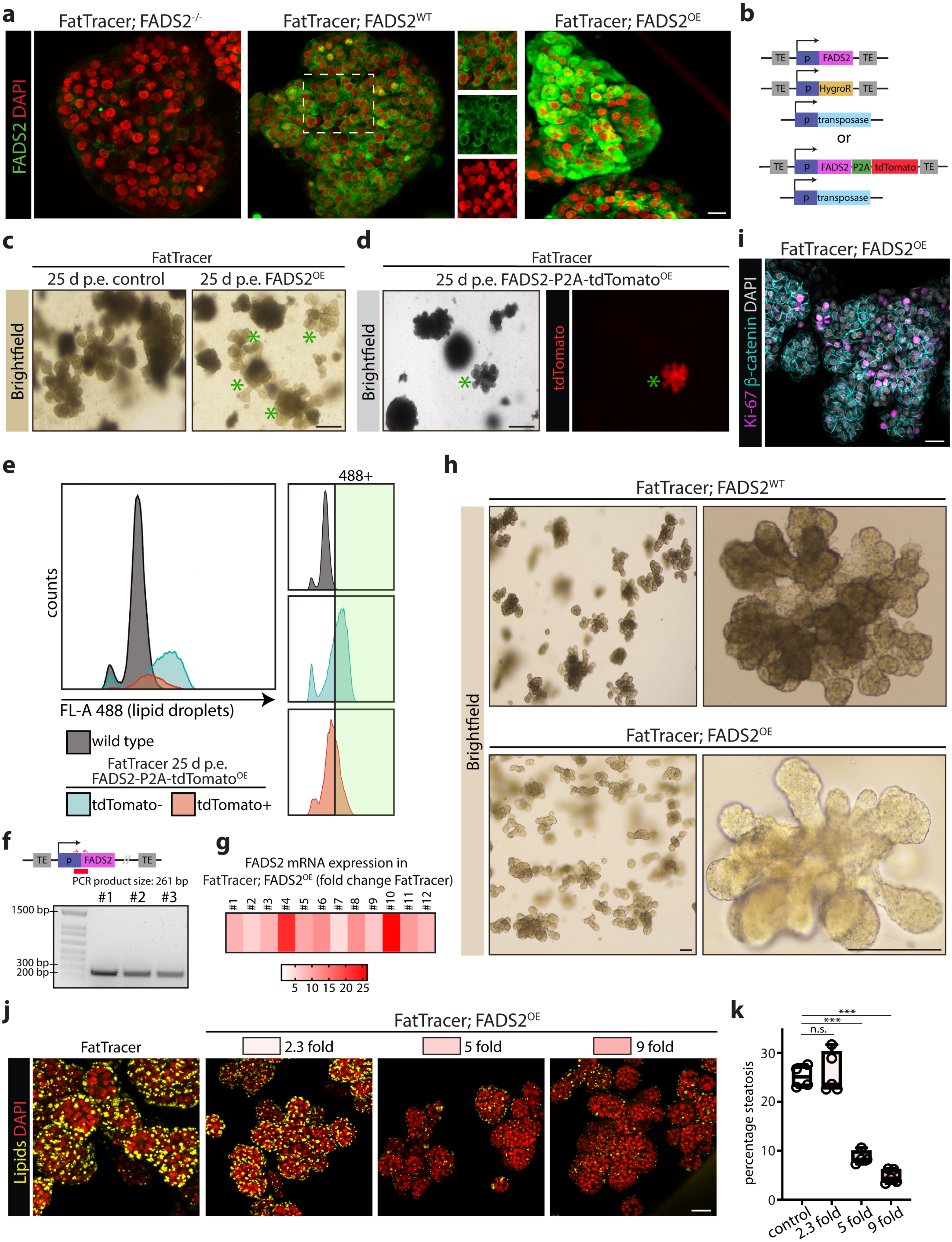
a
FatTracer; FADS2-/-
FatTracer; FADS2WT
FatTracer; FADS2OE
FADS2 DAPI
b
TE p FADS2 TE
TE p HygroR TE
p transposase
or
TE p FADS2 P2A tdTomato TE
p transposase
c
FatTracer
25 d p.e. control
25 d p.e. FADS2OE
Brightfield
d
FatTracer
25 d p.e. FADS2-P2A-tdTomatoOE
Brightfield
tdTomato
i
FatTracer; FADS2OE
Ki-67 β-catenin DAPI
e
488+
counts
FL-A 488 (lipid droplets)
wild type
FatTracer 25 d p.e.
FADS2-P2A-tdTomatoOE
tdTomato-
tdTomato+
h
FatTracer; FADS2WT
FatTracer; FADS2OE
Brightfield
f
TE p FADS2 TE
PCR product size: 261 bp
#1 #2 #3
1500 bp
300 bp
200 bp
g
FADS2 mRNA expression in
FatTracer; FADS2OE (fold change FatTracer)
#1 #2 #3 #4 #5 #6 #7 #8 #9 #10 #11 #12
5 10 15 20 25
j
FatTracer; FADS2OE
FatTracer
2.3 fold
5 fold
9 fold
Lipids DAPI
k
percentage steatosis
0 10 20 30
n.s.
***
***
control
2.3 fold
5 fold
9 fold

Supplementary Figure 14. FADS2 overexpression alleviates steatosis in FatTracer.

a, Immunofluorescence staining for FADS2 in FADS2 variant FatTracer organoids.

b, Schematic of the constructs used to overexpress FADS2 or FADS2-P2A-tdTomato using a transposon-based strategy.

c, Brightfield images of outgrowing FatTracer organoids upon transfection with the FADS2 overexpression construct. Asterisks highlight the appearance of lighter -less lipid containing- organoids.

d, Brightfield and red fluorescence images of outgrowing FatTracer organoids upon transfection with the FADS2-P2A-tdTomato overexpression construct. The asterisk highlights the appearance of a lighter -less lipid containing- organoid tdTomato$^+$ organoid.

e, FACS analysis of single cells from a FatTracer culture transfected with the FADS2-P2A-tdTomato construct 25 days p.e., as well as single cells from wild type organoids using the BioTracker 488 Green Lipid Dye, demonstrating that FADS2-overexpressing FatTracer cells (tdTomato$^+$) show reduced lipid droplet intensity (nearing wild type cells) as compared to FatTracer cells not overexpressing FADS2 (tdTomato$^-$).

f, Schematic representation of the PCR amplification area to confirm genomic integration of CAG-FADS2 (top) and gel results demonstrating integration of the construct with the expected size in different FatTracer; FADS2^{OE} lines (bottom).

g, Level of FADS2 mRNA overexpression in different FatTracer; FADS2^{OE} lines relative to the parental FatTracer lines generated from 2 different donors, as determined by qPCR analysis.

h, Brightfield images (low and high magnification) of FatTracer and FatTracer; FADS2^{OE} lines.

i, Immunofluorescence staining for Ki-67 and β-catenin in FatTracer; FADS2^{OE} organoids.

j, Nile Red lipid staining of FatTracer lines with various degrees of FADS2 overexpression, demonstrating that a threshold amount of FADS2 is needed for steatosis-reducing effects.

k, Quantification of the percentage of steatosis in FatTracer lines with various degrees of FADS2 overexpression in comparison to the original FatTracer line. n = 4 independent replicates for control and 5 fold conditions, n = 5 independent replicates for 2.3 fold and 9 fold conditions. Two-tailed *t*-test: $P < 0.0001$ (***); n.s. = not significant.

k, The box indicates the 25-75th percentiles, the centre line indicates the median and the whiskers indicate minimum and maximum values.

a, c, d, f, h, i, j, Representative of n = 2, 6, 6, 6, 6, 2, 3 independent experiments, respectively using both *APOB*$^{-/-}$ and *MTTP*$^{-/-}$ organoids as FatTracer from 2 donors.

Scale bars, 25 μm (**a**, **i**), 200 μm (**c**, **d**),100 μm (**h**), 50 μm (**j**).

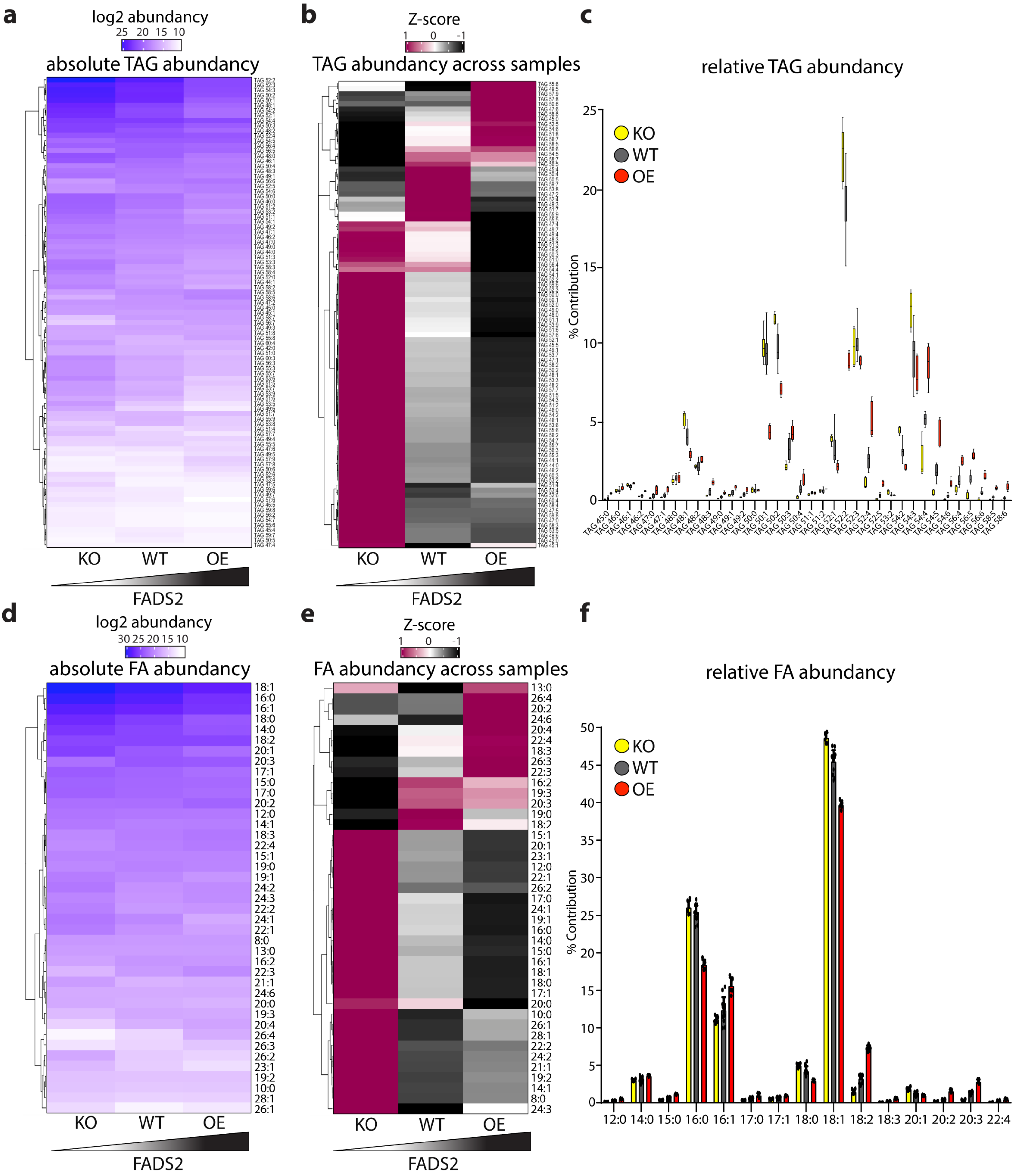

Supplementary Figure 15. Lipidomic analyses of FADS2 variant FatTracer organoids.

a, Heatmap displaying the average absolute abundancy (log2-transformed) of all detected TAG species in FADS2 variant FatTracer organoids. Data are derived from n = 6 independent measurements in 3 different clonal lines from 2 donors for FatTracer; FADS2$^{-/-}$ and FatTracer; FADS2^{OE}, n = 12 independent measurements in FatTracer; FADS2^{WT} from the same 2 donors.

b, Heatmap displaying the average TAG species abundancy in FADS2 variant FatTracer organoids. Row Z-scores are plotted.

c, Box plots depicting the relative TAG species abundancy within FADS2 variant FatTracer organoids.

d, Heatmap displaying the average absolute abundancy (log2-transformed) of all detected fatty acid (FA) species in TAG of FADS2 variant FatTracer organoids.

e, Heatmap displaying the average FA abundancy in TAG of FADS2 variant FatTracer organoids. Row Z-scores are plotted.

f, Bar plots depicting the relative FA abundancy in TAG within FADS2 variant FatTracer organoids (mean + SD).

b-f, Sample sizes as in **a**.

c, The box indicates the 25-75th percentiles, the centre line indicates the median and the whiskers indicate minimum and maximum values.

Supplementary Table 1. Donor information and NAFLD risk SNP characteristics of the human fetal hepatocyte organoid lines used in this study.

Donor	Age (GW)	Gender	PNPLA3<br>rs738409 (I148M)	TM6SF2<br>rs58542926 (E167K)	GCKR<br>rs1260326 (P446L)
A	12	F	I148I/I148I	E167E/E167E	P446P/P446P
B	8	M	I148I/I148I	E167E/E167E	P446P/**P446L**
C	14	M	I148I/I148I	E167E/E167E	P446P/P446P
D	20	F	I148I/I148I	E167E/E167E	P446P/P446P

Supplementary Table 2. Details of NAFLD drug candidate screening.

Drug	Target	Activation/ Inhibition	Concentrations tested	Vendor
Bempedoic acid	ATP citrate lyase (ACL)	Inhibition	5 – 10 – 25 – 50 μM	MedChemExpress
Firsocostat (GS-0976)	Acetyl-CoA carboxylase (ACC)	Inhibition	0.1 – 1 – 2.5 – 10 μM	SelleckChem
Cardarine (GW-501516)	Peroxisome proliferator-activated receptor δ (PPARδ)	Activation	0.1 – 1 – 2.5 – 10 μM	Sigma-Aldrich
TVB2640 (ASC-40)	Fatty acid synthase (FAS)	Inhibition	0.1 – 1 – 2.5 – 10 μM	InvivoChem
Aramchol	Stearoyl-CoA desaturase 1 (SCD1)	Inhibition	0.1 – 1 – 2.5 – 10 μM	Sigma-Aldrich
SRT2104	Sirtuin 1 (SIRT1)	Activation	0.1 – 1 – 2.5 – 10 μM	SelleckChem
Resmetirom (MGL-3196)	Thyroid hormone receptor β (THRβ)	Activation	0.1 – 1 – 2.5 – 10 μM	MedChemExpress
Cilofexor (GS-9674)	Farsenoid X receptor (FXR)	Activation	0.1 – 1 – 2.5 – 10 μM	MedChemExpress
Saroglitazar	Peroxisome proliferator-activated receptor α and γ (PPARα/γ)	Activation	5 – 10 – 25 – 50 μM	MedChemExpress
Fenofibrate	Peroxisome proliferator-activated receptor α (PPARα)	Activation	5 – 10 – 25 – 50 μM	Sigma-Aldrich
Elafibranor	Peroxisome proliferator-activated receptor α and δ (PPARα/δ)	Activation	5 – 10 – 25 – 50 μM	SelleckChem
Lanifibranor	Peroxisome proliferator-activated receptor α, γ, and δ (PPARα/γ/δ)	Activation	5 – 10 – 25 – 50 μM	SelleckChem
Pioglitazone	Peroxisome proliferator-activated receptor γ (PPARγ)	Activation	5 – 10 – 25 – 50 μM	Sigma-Aldrich
PF 06424439	Diacylglycerol O-acyltransferase 2 (DGAT2)	Inhibition	0.1 – 1 – 2.5 – 10 μM	Tocris
AZD 3988	Diacylglycerol O-acyltransferase 1 (DGAT1)	Inhibition	0.1 – 1 – 2.5 – 10 μM	Tocris
Recombinant hFGF19	β-Klotho/FGFRs	Activation	25 – 100 ng/ml	Peprotech
Recombinant hFGF21	β-Klotho/FGFRs	Activation	25 – 100 ng/ml	Peprotech

Supplementary Table 3. CRISPR sequences used in this study.

Gene knock-out

Plasmid name	Spacer sequence
ACACA-KO-sgRNA	CTTCCCTAATCTCTTCAGAC
ACACB-KO-sgRNA	GACAGATTTCTTACACTCCC
ACE2-KO-sgRNA	AGAACAGGTCTTCGGCTTCG
ACLY-KO-sgRNA	GAGCATACTTGAACCGATTC
ACSL1-KO-sgRNA	GAAGAGTACGCACGTACTGT
ACSS1-KO-sgRNA	TCCATGCCTCTTCAGCGTGT
ACSS2-KO-sgRNA	CATGCCCTGGCCCATTCCTT
AKR1C1-KO-sgRNA	TGTTACCTCTGCAGGCGCAT
AKR1C2-KO-sgRNA	ATCATTCAGCTTCACACAC
ALDOA-KO-sgRNA	TGACATCGCTCACCGCATCG
ANGPTL3-KO-sgRNA	TAATTTGGCCCTTCGTCTTA
APOB-KO-sgRNA	CAGCCAGTGCACCCTGAAAG
DGAT1-KO-sgRNA	GCGACCCTGTCCTCCGGCGC
DGAT2-KO-sgRNA	GACCTGCGCTGTCGCGCGAG
DHCR7-KO-sgRNA	TTGAGATGCGGTTCTGTCAT
FADS1-KO-sgRNA	GGCTGTCAGGCGCGTGCTCG
FADS2-KO-sgRNA	CCAGACTTACGTTCTTGCCG
FASN-KO-sgRNA	GGACAACCTCATCGGCGGTG
FDPS-KO-sgRNA	GATTCATCCCTTACCCGCCG
HK2-KO-sgRNA	GAAGTAGGCAAGCAGATGCG
LPCAT1-KO-sgRNA	CGCACCATGTGGTTCGCCGG
LSS-KO-sgRNA	GAACGGGATGACATTTTACG
MOGAT3-KO-sgRNA	GAAAGTGAGCACATATTGGT
MTARC1-KO-sgRNA	GTGCACTCCGCCTCGCTCAC
MTARC2-KO-sgRNA	TGGCTCGGGGTCGCCGCGCT
MTTP-KO-sgRNA	TGACCAGTTGATCCAAATAA
MVD-KO-sgRNA	GTGCAGAGTGACGCTCAGGG
PCKS9-KO-sgRNA	GGTGCTAGCCTTGCGTTCCG
PFKFB3-KO-sgRNA	TGTAGGTCTTGCCCCGGGCG
PKIB-KO-sgRNA	AAAATGACTGACGTGGAGTC
PNPLA3-KO-sgRNA	GCGCATGTTGTTCGGCGCTT
SCARB1-KO-sgRNA	CCTGCGGCTTCTCGCCCTTC
SLC23A3-KO-sgRNA	CCCAGCCAACTCCGATCAGT
SLC25A42-KO-sgRNA	ATGATTTTGGTTCGGTCCAG
SLC29A3-KO-sgRNA	AATAGTGGCCGTTGTCTCAG
SORBS1-KO-sgRNA	CTGGTTTGCTTTCGTGTTGC
SQLE-KO-sgRNA	CGAGGAGACCCCCGTTTCGG
TRIB3-KO-sgRNA	GCCCACTTCGAGCTCGTTTC

Prime editing

Plasmid name	Spacer sequence	3’- extension
PNPLA3-I148M-pegRNA	GGATAAGGCCACTGTAGAAG	GCTTCATGCCCTTCTACAGTGGCCTTA
PNPLA3-I148-PE3-sgRNA	AAGGATCAGGAAAATTAAAA	-
