# Supplementary material for: Engineered human hepatocyte organoids enable CRISPR-based target discovery and drug screening for steatosis
Source: Nat Biotechnol. 2023 Feb 23;41(11):1567–81. doi: 10.1038/s41587-023-01680-4 (PMC10635827; doi:10.1038/s41587-023-01680-4)
Supplement: Supplementary file 2 — Reporting Summary [file 41587_2023_1680_MOESM2_ESM.pdf]

nature research

Corresponding author(s): Delilah Hendriks, Benedetta Artegiani, Hans Clevers

Last updated by author(s): Dec 15, 2022

# Reporting Summary

Nature Research wishes to improve the reproducibility of the work that we publish. This form provides structure for consistency and transparency in reporting. For further information on Nature Research policies, see our Editorial Policies and the Editorial Policy Checklist.

## Statistics

For all statistical analyses, confirm that the following items are present in the figure legend, table legend, main text, or Methods section.

| n/a | Confirmed | |
|---|---|---|
| ☐ | ☒ | The exact sample size (*n*) for each experimental group/condition, given as a discrete number and unit of measurement |
| ☐ | ☒ | A statement on whether measurements were taken from distinct samples or whether the same sample was measured repeatedly |
| ☐ | ☒ | The statistical test(s) used AND whether they are one- or two-sided<br>*Only common tests should be described solely by name; describe more complex techniques in the Methods section.* |
| ☒ | ☐ | A description of all covariates tested |
| ☒ | ☐ | A description of any assumptions or corrections, such as tests of normality and adjustment for multiple comparisons |
| ☐ | ☒ | A full description of the statistical parameters including central tendency (e.g. means) or other basic estimates (e.g. regression coefficient) AND variation (e.g. standard deviation) or associated estimates of uncertainty (e.g. confidence intervals) |
| ☐ | ☒ | For null hypothesis testing, the test statistic (e.g. *F*, *t*, *r*) with confidence intervals, effect sizes, degrees of freedom and *P* value noted<br>*Give P values as exact values whenever suitable.* |
| ☒ | ☐ | For Bayesian analysis, information on the choice of priors and Markov chain Monte Carlo settings |
| ☒ | ☐ | For hierarchical and complex designs, identification of the appropriate level for tests and full reporting of outcomes |
| ☐ | ☒ | Estimates of effect sizes (e.g. Cohen's *d*, Pearson's *r*), indicating how they were calculated |

*Our web collection on statistics for biologists contains articles on many of the points above.*

## Software and code

Policy information about availability of computer code

| | |
|---|---|
| Data collection | Leica LAS X (v1.1), X500R QToF type mass spectrometer software, Illumina NextSeq2000 software. |
| Data analysis | Fiji (v2.0.0), Leica LAS X (v1.1), Microsoft Excel 2020, GraphPad Prism (v8.2.0), RStudio (v2022.02.2), R (v4.2.0), Photoshop CS4. |

For manuscripts utilizing custom algorithms or software that are central to the research but not yet described in published literature, software must be made available to editors and reviewers. We strongly encourage code deposition in a community repository (e.g. GitHub). See the Nature Research guidelines for submitting code & software for further information.

## Data

Policy information about availability of data

All manuscripts must include a data availability statement. This statement should provide the following information, where applicable:

- Accession codes, unique identifiers, or web links for publicly available datasets
- A list of figures that have associated raw data
- A description of any restrictions on data availability

RNA sequencing data is deposited in Gene Expression Omnibus under accession number GSE221705.



April 2020

# Field-specific reporting

Please select the one below that is the best fit for your research. If you are not sure, read the appropriate sections before making your selection.

☒ Life sciences ☐ Behavioural & social sciences ☐ Ecological, evolutionary & environmental sciences

For a reference copy of the document with all sections, see nature.com/documents/nr-reporting-summary-flat.pdf

# Life sciences study design

All studies must disclose on these points even when the disclosure is negative.

| | |
|---|---|
| Sample size | No sample size calculation was performed to predetermine sample sizes. The samples sizes used were in the same range of other studies in the field. Results were validated across organoid lines from at least 2 different donors. |
| Data exclusions | No datapoints were excluded. |
| Replication | Experiments were confirmed across multiple independent experiments, across multiple organoids, and across organoids from different donors. All attempts of replication were successful. Detailed information about replicate numbers are given in the figure legends. |
| Randomization | Not applicable. There are no experiments that require randomization and therefore no randomization was performed. |
| Blinding | The researchers were not blinded since no experiments needed blinding for analysis. |

# Reporting for specific materials, systems and methods

We require information from authors about some types of materials, experimental systems and methods used in many studies. Here, indicate whether each material, system or method listed is relevant to your study. If you are not sure if a list item applies to your research, read the appropriate section before selecting a response.

## Materials & experimental systems

| n/a | Involved in the study | |
|---|---|---|
| ☐ | ☒ | Antibodies |
| ☐ | ☒ | Eukaryotic cell lines |
| ☒ | ☐ | Palaeontology and archaeology |
| ☒ | ☐ | Animals and other organisms |
| ☐ | ☒ | Human research participants |
| ☒ | ☐ | Clinical data |
| ☒ | ☐ | Dual use research of concern |

## Methods

| n/a | Involved in the study | |
|---|---|---|
| ☒ | ☐ | ChIP-seq |
| ☒ | ☐ | Flow cytometry |
| ☒ | ☐ | MRI-based neuroimaging |

## Antibodies

**Antibodies used**

Primary: mouse anti-ApoB (989529), MAB41241-SP, Novus Biologicals, dilution 1:50; rabbit anti-beta-catenin (H-102), sc-7199, Santa Cruz, dilution 1:200; rabbit anti-FADS2/Delta-6 Desaturase (aa79-108), LS-C165916, LSBio, dilution 1:50; rat anti-Ki-67 (SolA15), 14-5698-82, Thermo Fisher; dilution 1:1000; rabbit anti-MTP, ab63467, Abcam, dilution 1:500; sheep anti-PNPLA3/Adiponutrin, AF5208, R&D systems, dilution 1:100.
Secondary: Alexa Fluor 488 anti-mouse, A11029; Alexa Fluor 488 anti-rabbit, A21206; Alexa Fluor 568 anti-sheep, A21099; Alexa Fluor 568 anti-rabbit; Alexa Fluor 647 anti-rat, A21247; Alexa Fluor 647 anti-rabbit A21245. All secondary antibodies were purchased from Thermo Fisher, dilution 1:1000.
Cell membranes were stained with Phalloidin-Atto 647N, 65906, Sigma-Aldrich, dilution 1:2000.

**Validation**

All antibodies were validated by the manufacturers and have been used across multiple publications.

Anti-Ki-67 purified (SolA15) #14-5698-82 (https://www.thermofisher.com/antibody/product/Ki-67-Antibody-clone-SolA15-Monoclonal/14-5698-82). This Antibody was verified by Cell treatment to ensure that the antibody binds to the antigen stated.
Citations: e.g. PMID: 34525348, PMID: 34100459

Anti-APOB #MAB41241-SP (https://www.bio-techne.com/p/antibodies/human-apolipoprotein-b-apob-antibody-989529_mab41241). Apolipoprotein B/ApoB in Human HepG2 and MCF-7 Cell Lines. Apolipoprotein B/ApoB was detected in immersion fixed HepG2 human hepatocellular carcinoma cell line (positive control) and MCF-7 human breast cancer cell line (negative control) using Mouse Anti-Human Apolipoprotein B/ApoB Monoclonal Antibody (Catalog # MAB41241) at 8 µg/mL for 3 hours at room temperature.

Anti-MTTP #ab63467 (https://www.abcam.com/mttpmtp-antibody-ab63467.html). This antibody gave a positive signal in Human Liver Tissue Lysate.
Citations: e.g. PMID: 29074218, PMID: 28959527, PMID: 22648712.

Phalloidin–Atto 647N #65906 (https://www.sigmaaldrich.com/NL/en/product/sigma/65906).
Citations: e.g. PMID: 32123335.

Anti-FADS2/delta-6 desaturase (aa79-108) (https://www.lsbio.com/antibodies/delta-6-desaturase-antibody-fads2-antibody-aa79-108-flow-if-immunofluorescence-ihc-wb-western-ls-c165916/173263#reviews-section). FADS2 antibody LS-C165916 is an unconjugated rabbit polyclonal antibody to human FADS2 (Delta-6 Desaturase) (aa79-108). Validated for Flow, IF, IHC and WB.

Anti-PNPLA3/adiponutrin #AF5208. (https://www.rndsystems.com/products/human-adiponutrin-pnpla3-antibody_af5208).
Adiponutrin/ADPN was detected in immersion fixed human adipocytes using 10 µg/mL Sheep Anti-Human Adiponutrin/ADPN Antigen Affinity-purified Polyclonal Antibody (Catalog # AF5208) for 3 hours at room temperature.
Citations: e.g. PMID34288010.

## Eukaryotic cell lines

Policy information about cell lines

| | |
|---|---|
| Cell line source(s) | All human hepatocyte organoid lines used in this study were derived from human fetal liver tissues from termination material from donors with informed consent under ethical permission (Leiden University Medical Center). |
| Authentication | None of the organoid lines were authenticated. |
| Mycoplasma contamination | All organoid lines were regularly assessed for mycoplasma contamination and scored negatively without exception. |
| Commonly misidentified lines (See ICLAC register) | N.A. |

## Human research participants

Policy information about studies involving human research participants

| | |
|---|---|
| Population characteristics | Anonymized healthy human fetal livers from both males and females were included. |
| Recruitment | We included in the study any available anonymously donated healthy human fetal liver tissues upon informed consent. No pre-selection was performed, to avoid any bias. |
| Ethics oversight | Anonymized human fetal livers became available after pregnancy terminations and upon informed consent and under ethical permission from the Leiden University Medical Center. |

Note that full information on the approval of the study protocol must also be provided in the manuscript.
